# Social mobility and cancer mortality in Central and Eastern Europe: a multicohort study

**DOI:** 10.1093/eurpub/ckag124

**Published:** 2026-07-11

**Authors:** Yuwei Pan, Hynek Pikhart, Andrzej Pajak, Abdonas Tamosiunas, Tatyana Court, Magdalena Kozela, Anne Peasey, Denes Stefler, Martin Bobak

**Affiliations:** Research Department of Epidemiology and Public Health, University College London, London, United Kingdom; Research Department of Epidemiology and Public Health, University College London, London, United Kingdom; Recetox, Faculty of Science, Masaryk University, Brno, Czech Republic; Department of Epidemiology and Population Studies, Institute of Public Health, Faculty of Health Sciences, Jagiellonian University Medical College, Krakow, Poland; Institute of Cardiology, Medical Academy, Lithuanian University of Health Sciences, Kaunas, Lithuania; Recetox, Faculty of Science, Masaryk University, Brno, Czech Republic; Department of Epidemiology and Population Studies, Institute of Public Health, Faculty of Health Sciences, Jagiellonian University Medical College, Krakow, Poland; Research Department of Epidemiology and Public Health, University College London, London, United Kingdom; Research Department of Epidemiology and Public Health, University College London, London, United Kingdom; Research Department of Epidemiology and Public Health, University College London, London, United Kingdom; Recetox, Faculty of Science, Masaryk University, Brno, Czech Republic

## Abstract

Social determinants of cancer mortality in Central and Eastern Europe (CEE) have been relatively under-researched. This study investigated associations of socioeconomic status (SES) and intergenerational social mobility with cancer mortality in three countries of the region. Data from the Health, Alcohol and Psychosocial factors In Eastern Europe (HAPIEE) study in the Czech Republic, Poland, and Lithuania were used (23 009 men and women, an average follow-up of 17 years, and a total of 2383 cancer deaths through the end of 2024). Childhood SES was measured by parental education and household amenities at age 10, and adulthood SES was measured by education, employment, household amenities, and material deprivation. Principal component analysis based on polychoric correlation matrix reduced the dimensionality of SES variables. Competing risk models estimated subdistribution hazard ratios (SHRs) for the associations of SES and social mobility with mortality from all cancers and two leading cancer types (lung and colorectal cancers). Compared to low adulthood SES, high adulthood SES was associated with lower total and lung cancer mortality (fully adjusted SHRs were 0.85 (95% confidence interval [CI], 0.76-0.94) and 0.79 (0.62-1.00), respectively). Compared to stable low SES, stable middle or high SES was associated with lower overall cancer mortality (fully adjusted SHR was 0.88 [95% CI, 0.77-1.00]), while associations of downward and upward mobility with cancer mortality varied by cancer type and country. Adulthood SES and intergenerational social mobility, but not childhood SES, were associated with cancer mortality in later life in CEE. These results may help inform cancer prevention and treatment programmes.

## Introduction

Cancer accounts for over 22% of total deaths in Europe in 2022 [1], with pronounced regional disparities [2–4] and highest mortality rates in Central and Eastern Europe (CEE) [2]. Irrespective of country, European residents with lower adulthood socioeconomic status (SES) were at higher risk of death from most types of cancer compared to their counterparts with higher SES [5–10], with lung cancer being the largest contributor to this inequality [5, 11]. A potential exception is the inverse social gradient in breast cancer [9, 12]. Overall, this socioeconomic inequality, measured by education, was generally greater in CEE and smaller in South-Europe [5], especially among men [5, 11].

Most studies examined the association between adulthood SES and cancer, but more recently, there has been increasing interest in influences of childhood SES and life course SES in general [13–20]. Existing evidence suggests that the effects of childhood SES (usually measured by parental education or occupation or household material status in childhood) differ by the measurement of cancer (morbidity or mortality) and cancer type. When measured by parental occupation, high childhood SES appeared associated with higher later-life cancer prevalence [13], a higher risk of incident colon cancer in women [14], and a higher risk of incident cancer overall [15]. Not surprisingly, the associations between childhood SES and cancer mortality are often attenuated by controlling for adulthood SES [17, 19], especially for education, which might be related to socially patterned risky health behaviours [19]. Existing evidence on social mobility and cancer mortality is less complete and less consistent. While stable low SES is generally associated with higher cancer mortality, the effects of upward and downward mobility vary across studies [18, 21, 22]. Findings differ by country, sex, and whether intergenerational or intragenerational mobility is examined. Table S1 summarizes the key characteristics of the cited studies on SES and cancer.

Social mobility and cancer mortality have not been examined in CEE. In fact, there is still a relative lack of detailed evidence on SES and cancer from individual-based longitudinal studies in CEE, as most previous studies were based on routine data. To fill these gaps, this research examined the independent associations of childhood SES, adulthood SES, and intergenerational social mobility with mortality from all cancers and leading cancer types (lung and colorectal cancers), using data from a prospective multi-cohort study in three countries of CEE.

## Methods

### Study population

Data from the Czech, Polish, and Lithuanian arms of the Health, Alcohol and Psychosocial factors In Eastern Europe (HAPIEE) study were used [23]. The baseline survey was conducted in 2002–2005 for the Czech Republic and Poland, and in 2006–2008 for Lithuania, and collected information on socioeconomic circumstances, health, lifestyle, diet, psychosocial factors, anthropometrics, and blood biomarkers. A total of 26 745 men and women aged 45–75 from these three countries were interviewed at baseline. Follow-up information on mortality was updated until December 2024 for Czech Republic and Lithuania, and December 2021 for Poland. This study was approved by the ethics committee at University College London in the UK and the ethics committee in each participating centre. Written informed consent was obtained from all participants.

### Sample selection

After excluding 716 Polish participants who did not agree to follow-up, 26 029 participants were eligible. Among them, 2627 participants with missing data in childhood and/or adulthood SES and 393 participants with missing data in covariates were further excluded. Therefore, the analytical sample comprised 23 009 complete cases (Czech Republic: *n* = 7545, Poland: *n* = 9252, and Lithuania: *n* = 6212). Fig. S1 illustrates the sample selection procedure.

### SES measurement

Childhood SES was measured at baseline using (i) parental education (Incomplete primary or lower; Incomplete secondary or primary; Secondary or vocational; University or college) and/or (ii) household amenities at age 10 (divided into Low [0–2 items], Medium [3–4 items], and High [5 or 6 items] based on 6 items including cold tap water, hot tap water, a radio, a fridge, own kitchen, own toilet). Since parental education was asked only in subsample of Czech participants, many Czech participants had missing data on this variable. Therefore, we created an alternative classification of childhood SES in full Czech sample using only household amenities at age 10; the comparison of the two classifications is shown in Table S2. In Poland and Lithuania, childhood SES was measured by both parental education and household amenities at age 10.

Adulthood SES was measured at baseline using four domains: (i) employment status (employed/entrepreneur/self-employed/freelance/other [housewife, farmer, employed pensioner]/not employed pensioner/unemployed); (ii) education (primary or lower; secondary or vocational; university or college); (iii) number of household amenities (divided into low [less than 5 items], medium [5–8 items], and high [9–12 items] based on 12 items microwave, video recorder, colour TV, washing machine, dishwasher, car, freezer, cottage, video camera, satellite/cable TV, telephone, mobile phone) in Czech Republic and Poland, and 10 items in Lithuania; and (iv) absolute material deprivation (frequency of difficulties in paying bills, buying food and buying clothes necessary for the subject and/or his family, divided into low [‘all the time’/‘often’ have difficulties in any of the 3 items], medium [‘sometimes’/‘rarely’ in any of the 3 items], and high [‘never’ have difficulty in all 3 items]). Principal component analysis based on polychoric correlation matrix was used to reduce the dimensionality of SES variables [24]. Both childhood SES and adulthood SES were categorized into (i) low, (ii) middle, and (iii) high. Intergenerational social mobility was defined based on childhood and adulthood SES, and it was categorized into (i) stable low SES, (ii) downward mobility (middle or high childhood SES but low adulthood SES), (iii) upward mobility (low childhood SES and middle or high adulthood SES), and (iv) stable middle or high SES.

### Cancer mortality rate

Mortality information, including alive status, date of death, and cause of death, was from national death register in the Czech Republic (2002–2024), provincial death register in Poland (2002–2021), and regional mortality register in Lithuania (2005–2024); mortality registers in these countries are considered complete [25]. Cancer mortality included overall-cancer mortality (ICD-10: C00-C97), lung cancer mortality (ICD-10: C34), and colorectal cancer mortality (ICD-10: C18-20).

### Covariates

Demographic factors included age (years), sex (male; female), and marital status (single; married or cohabiting; divorced, separated, or widowed). Health behaviours included smoking status (categorized as never smoker; former smoker; current smoker) and daily alcohol consumption (including consumption of beer, wine, and spirits, measured by average gram per day).

### Statistical methods

Chi-squared test, chi-squared test for trend, one-way analysis of variance (ANOVA), and Kruskal–Wallis test were used to investigate the associations of covariates with childhood or adulthood SES, social mobility, and cancer mortality (Tables S3 and S4). Since death from other causes would preclude death from cancer and alter the probability of occurrence of death from cancer, competing-risks regression based on Fine and Gray’s proportional subdistribution hazards model was applied to investigate associations of childhood SES, adulthood SES, and social mobility with cancer mortality [26]. For deaths from any cancer, the competing causes were non-cancer deaths, while for lung and colorectal cancers, the competing events were deaths from any other cause including other cancer types. Models were estimated in all three countries together and in each country separately. Participants who remained alive by the end of follow-up were censored. Proportional subdistribution hazards assumption was tested by including time interactions on covariates. Estimated subdistribution hazard ratios (SHRs) and corresponding 95% confidence intervals (CIs) from age- and sex-adjusted model and fully adjusted model were presented in the main tables; sex-specific results are presented in Tables S5–S9.

Covariates were added to the model using a forward stepwise strategy. Interaction terms between social mobility and covariates were used to identify potential effect modification. Cumulative incidence function (CIF), which estimated the marginal probability of death from cancer, was plotted following the estimation of competing risk models. Furthermore, to make direct comparison between countries and to show the absolute differences, age-standardized cancer mortality rates (ASMRs) were calculated using the European Standard Population 2013 [27]. Sensitivity analyses were conducted to investigate whether the analytical sample differed significantly from the excluded participants, and the effect of childhood SES measured by both parental education and household amenities at age 10 in Czech Republic. All the analyses were performed using StataNow/MP version 19.5.

## Results

### Sample characteristics

Table 1 shows the baseline sample characteristics by country, as well as the corresponding cancer mortality rates. Across the three countries, 40% of the participants consistently belonged to the middle or high SES, 25% experienced an upward mobility, 21% experienced a downward mobility, and 14% remained in low SES. Over an average follow-up time of 17 years, 15 774 (69%) participants remained alive, 2383 (10%) participants died from cancer, and 4852 (21%) participants died from other causes. The overall cancer mortality rate was 6.22 per 1000 person-years in the three CEE countries (Czech Republic: 6.05 per 1000 person-years, Poland: 6.37 per 1000 person-years, Lithuania: 6.22 per 1000 person-years). Generally, total cancer mortality rate was higher in males, divorced, separated, or widowed participants, current smokers, and participants with lower childhood SES or adulthood SES. Table S10 presents the cancer mortality rate (per 1000 person-years) by childhood SES, adulthood SES, and social mobility groups in each country. Table S11 compared the baseline characteristics of the analytical sample and the excluded participants; significant difference was only observed in marital status, with a 4.6% higher proportion of married participants in the analytical sample than the excluded. All baseline covariates were associated with social mobility and adulthood SES and, except for sex, with childhood SES, and all covariates except for marital status were associated with total cancer mortality, lung cancer mortality, and colorectal cancer mortality (Tables S3 and S4).

**Table 1.** Baseline sample characteristics according to country of residence

Sample characteristics		*N* (col %)				*N* of death from cancer	Cancer mortality rate per 1000 person-years
		Total	Czech Republic	Poland	Lithuania		
		*n* = 23 009	*n* = 7545 (row%=32.79)	*n* = 9252 (row%=40.21)	*n* = 6212 (row%=27.00)		
Age (years)	Mean (SD)	58.63 (7.35)	58.03 (7.16)	57.60 (6.97)	60.90 (7.62)	2383	6.22
Sex	Male	10 899 (47.37)	3572 (47.34)	4514 (48.79)	2813 (45.28)	1403	8.12
	Female	12 110 (52.63)	3973 (52.66)	4738 (51.21)	3399 (54.72)	980	4.65
Marital status	Single	919 (3.99)	194 (2.57)	483 (5.22)	242 (3.90)	85	5.75
	Married/cohabiting	17 240 (74.93)	5754 (76.26)	7103 (76.77)	4383 (70.56)	1788	6.17
	Divorced/separated/widowed	4850 (21.08)	1597 (21.17)	1666 (18.01)	1587 (25.55)	510	6.45
Smoking status	Never smoker	10 859 (47.19)	3291 (43.62)	3644 (39.39)	3924 (63.17)	848	4.55
	Former smoker	6021 (26.17)	2257 (29.91)	2626 (28.38)	1138 (18.32)	680	6.88
	Current smoker	6129 (26.64)	1997 (26.47)	2982 (32.23)	1150 (18.51)	855	8.69
Daily alcohol consumption (g/day)	Mean (SD)	162.97 (367.15)	287.26 (465.87)	122.91 (314.87)	71.67 (242.01)	2383	6.22
Household amenities at age 10	High (5 or 6 items)	6785 (29.49)	2955 (39.17)	3093 (33.43)	737 (11.86)	554	4.58
	Medium (3–4 items)	9559 (41.54)	942 (12.49)	3537 (38.23)	2186 (35.19)	1026	6.45
	Low (0–2 items)	6665 (28.97)	3648 (48.35)	2622 (28.34)	3289 (52.95)	803	7.76
Father’s education^a^	University/college	2041 (13.20)	–	1030 (11.13)	1011 (16.27)	191	5.84
	Secondary/vocational	4423 (28.60)		3547 (38.34)	876 (14.10)	404	5.63
	Incomplete secondary/primary	7323 (47.36)		3895 (42.10)	3428 (55.18)	758	6.60
	Incomplete primary or lower	1677 (10.84)		780 (8.43)	897 (14.44)	187	7.55
Mother’s education^a^	University/college	1128 (7.29)	–	401 (4.33)	727 (11.70)	92	5.01
	Secondary/vocational	3971 (25.68)		3127 (33.80)	844 (13.59)	363	5.60
	Incomplete secondary/primary	8283 (53.56)		4796 (51.84)	3487 (56.13)	844	6.48
	Incomplete primary or lower	2082 (13.46)		928 (10.03)	1154 (18.58)	241	7.86
Childhood SES	High	6955 (30.23)	2955 (39.17)	2715 (29.35)	1285 (20.69)	608	4.95
	Middle	7215 (31.36)	3648 (48.35)	2558 (27.65)	1009 (16.24)	767	6.22
	Low	8839 (38.42)	942 (12.49)	3979 (43.01)	3918 (63.07)	1008	7.34
Employment status	Employed/self-employed	10 759 (46.76)	3420 (45.33)	3453 (37.32)	3032 (48.81)	648	3.64
	Other	2345 (10.19)	655 (8.68)	769 (8.31)	921 (14.83)	267	7.09
	Not employed pensioner/unemployed	9905 (43.05)	3470 (45.99)	5030 (54.37)	2259 (36.37)	1468	8.75
Education	University/college	7396 (32.14)	1135 (15.04)	2650 (28.64)	3611 (58.13)	619	5.03
	Secondary/vocational	12 998 (56.49)	5533 (73.33)	5569 (60.19)	1896 (30.52)	1428	6.49
	Primary or lower	2615 (11.37)	877 (11.62)	1033 (11.17)	705 (11.35)	336	8.37
Number of households amenities^b^	High (9 to 12 items)	4939 (21.47)	1840 (24.39)	1732 (18.72)	1367 (22.01)	417	4.80
	Medium (5 to 8 items)	13 735 (59.69)	4471 (59.26)	5668 (61.26)	3596 (57.89)	1390	6.03
	Low (less than 5 items)	4335 (18.84)	1234 (16.36)	1852 (20.02)	1249 (20.11)	576	8.73
Absolute material deprivation	Low	3075 (13.36)	844 (11.19)	1752 (18.94)	479 (7.71)	333	6.75
	Medium	7310 (31.77)	2917 (38.66)	3040 (32.86)	1353 (21.78)	743	5.97
	High	12 624 (54.87)	3784 (50.15)	4460 (48.21)	4380 (70.51)	1307	6.24
Adulthood SES	High	7825 (34.01)	2218 (29.40)	2604 (28.15)	3003 (48.34)	668	4.96
	Middle	7196 (31.27)	2314 (30.67)	2807 (30.34)	2075 (33.40)	824	7.06
	Low	7988 (34.72)	3013 (39.93)	3841 (41.52)	1134 (18.25)	891	6.74
Social mobility	Stable middle or high SES	9263 (40.26)	4033 (53.45)	3243 (35.05)	1987 (31.99)	855	5.29
	Upward mobility	5758 (25.02)	499 (6.61)	2168 (23.43)	3091 (49.76)	637	7.12
	Downward mobility	4907 (21.33)	2570 (34.06)	2030 (21.94)	307 (4.94)	520	6.16
	Stable low SES	3081 (13.39)	443 (5.87)	1811 (19.57)	827 (13.31)	371	7.78
Status^c^	Alive	15 774 (68.56)	4943 (65.51)	6610 (71.44)	4221 (67.95)	–	–
	Death from lung cancer	497 (2.16)	177 (2.35)	228 (2.46)	92 (1.48)		
	Death from colorectal cancer	297 (1.29)	112 (1.48)	110 (1.19)	75 (1.21)		
	Death from other cancers	1589 (6.91)	554 (7.34)	611 (6.60)	424 (6.83)		
	Death from other causes	4695 (20.41)	1714 (22.72)	1588 (17.16)	1393 (22.42)		
	Death from unknown causes	157 (0.68)	45 (0.60)	105 (1.13)	7 (0.11)		

a Total number of participants with parental education is 15 464.

b Czech Republic and Poland:12 items; Lithuania:10 items.

c Death from cancer in all countries: *n* = 2383 (10.36%); Czech Republic: *n* = 843 (11.17%); Poland: *n* = 949 (10.26%); Lithuania: *n* = 591 (9.51%).

SD: standard deviation; SES: socioeconomic status.

### Individual SES and intergenerational social mobility

After controlling for age and sex, higher adulthood SES was associated with lower subdistribution hazard (subhazard) of total cancer death, compared to low adulthood SES, in all three countries (Table 2). Upward social mobility and stable middle or high SES were associated with lower subhazard. Further adjustment for marital status and health behaviours moderately attenuated these associations. There was insufficient evidence for the association between childhood SES and cancer mortality. The associations differed slightly when stratified by country. The predicted cumulative incidence of cancer death by social mobility, corresponding to the fully adjusted estimates shown in Table 2, is shown in Fig. S2.

**Table 2.** Associations of childhood SES, adulthood SES, and social mobility with cancer mortality in Central and Eastern Europe: subdistribution hazard ratios and 95% confidence intervals

	Subdistribution hazard ratios (95% confidence intervals)							
	All countries		Czech Republic		Poland		Lithuania	
	*n* = 23 009		*n* = 7545		*n* = 9252		*n* = 6212	
	Minimally adjusted^a^	Fully adjusted^b^	Age-sex adjusted	Fully adjusted^b^	Age-sex adjusted	Fully adjusted^b^	Age-sex adjusted	Fully adjusted^b^
(A) Total cancer								
Childhood SES								
Low	Ref	Ref	Ref	Ref	Ref	Ref	Ref	Ref
Middle	0.95 (0.86, 1.06)	0.92 (0.83, 1.02)	0.91 (0.75, 1.11)	0.90 (0.74, 1.09)	0.96 (0.82, 1.13)	0.92 (0.79, 1.07)	1.02 (0.81, 1.29)	1.00 (0.79, 1.26)
High	1.06 (0.95, 1.19)	1.02 (0.91, 1.15)	1.13 (0.90, 1.41)	1.11 (0.89, 1.38)	1.03 (0.87, 1.22)	0.97 (0.82, 1.15)	1.05 (0.83, 1.32)	1.04 (0.82, 1.31)
Adulthood SES								
Low	Ref	Ref	Ref	Ref	Ref	Ref	Ref	Ref
Middle	0.88 (0.80, 0.97)	0.93 (0.85, 1.03)	0.87 (0.74, 1.02)	0.91 (0.78, 1.07)	0.88 (0.76, 1.02)	0.94 (0.81, 1.09)	0.92 (0.73, 1.15)	0.94 (0.75, 1.18)
High	0.79 (0.71, 0.87)	0.85 (0.76, 0.94)	0.83 (0.69, 0.99)	0.88 (0.74, 1.06)	0.75 (0.64, 0.89)	0.82 (0.70, 0.98)	0.78 (0.62, 0.98)	0.82 (0.65, 1.04)
Social mobility								
Stable low SES	Ref	Ref	Ref	Ref	Ref	Ref	Ref	Ref
Downward mobility	1.06 (0.92, 1.22)	1.00 (0.87, 1.15)	1.13 (0.86, 1.50)	1.09 (0.82, 1.44)	1.00 (0.82, 1.22)	0.93 (0.76, 1.13)	1.22 (0.82, 1.84)	1.17 (0.78, 1.76)
Upward mobility	0.87 (0.77, 1.00)	0.92 (0.80, 1.05)	1.06 (0.76, 1.49)	1.10 (0.79, 1.54)	0.82 (0.68, 0.98)	0.87 (0.72, 1.05)	0.89 (0.69, 1.13)	0.92 (0.72, 1.17)
Stable middle or high SES	0.86 (0.76, 0.98)	0.88 (0.77, 1.00)	0.92 (0.70, 1.21)	0.94 (0.72, 1.24)	0.83 (0.69, 0.99)	0.85 (0.71, 1.01)	0.90 (0.68, 1.19)	0.91 (0.69, 1.21)
(B) Lung cancer								
Childhood SES								
Low	Ref	Ref	Ref	Ref	Ref	Ref	Ref	Ref
Middle	1.06 (0.85, 1.33)	0.96 (0.77, 1.20)	1.13 (0.72, 1.79)	1.07 (0.68, 1.70)	1.27 (0.93, 1.74)	1.13 (0.83, 1.55)	0.59 (0.29, 1.17)	0.54 (0.27, 1.08)
High	1.28 (1.00, 1.63)	1.16 (0.91, 1.47)	1.59 (0.95, 2.68)	1.52 (0.91, 2.56)	1.26 (0.90, 1.78)	1.12 (0.80, 1.57)	0.99 (0.56, 1.76)	0.96 (0.55, 1.70)
Adulthood SES								
Low	Ref	Ref	Ref	Ref	Ref	Ref	Ref	Ref
Middle	0.80 (0.65, 0.98)	0.91 (0.73, 1.12)	0.72 (0.50, 1.03)	0.81 (0.56, 1.17)	0.82 (0.61, 1.10)	0.93 (0.69, 1.25)	0.83 (0.48, 1.45)	0.98 (0.56, 1.73)
High	0.67 (0.53, 0.84)	0.79 (0.62, 1.00)	0.84 (0.59, 1.21)	0.97 (0.67, 1.40)	0.59 (0.42, 0.84)	0.69 (0.48, 0.98)	0.56 (0.31, 1.00)	0.79 (0.44, 1.42)
Social mobility								
Stable low SES	Ref	Ref	Ref	Ref	Ref	Ref	Ref	Ref
Downward mobility	1.33 (0.98, 1.80)	1.15 (0.85, 1.56)	1.26 (0.67, 2.35)	1.15 (0.61, 2.14)	1.57 (1.06, 2.35)	1.34 (0.90, 2.00)	0.53 (0.16, 1.81)	0.44 (0.13, 1.55)
Upward mobility	0.83 (0.61, 1.12)	0.93 (0.69, 1.27)	0.76 (0.34, 1.71)	0.82 (0.36, 1.86)	0.86 (0.57, 1.28)	0.96 (0.64, 1.44)	0.62 (0.35, 1.10)	0.78 (0.44, 1.39)
Stable middle or high SES	0.90 (0.67, 1.20)	0.93 (0.69, 1.23)	0.96 (0.52, 1.78)	1.01 (0.55, 1.87)	0.95 (0.64, 1.41)	0.96 (0.65, 1.41)	0.54 (0.28, 1.06)	0.66 (0.33, 1.29)
(C) Colorectal cancer								
Childhood SES								
Low	Ref	Ref	Ref	Ref	Ref	Ref	Ref	Ref
Middle	0.77 (0.58, 1.02)	0.76 (0.57, 1.01)	0.88 (0.54, 1.43)	0.88 (0.54, 1.43)	0.69 (0.42, 1.12)	0.68 (0.42, 1.11)	0.64 (0.31, 1.33)	0.63 (0.31, 1.30)
High	0.80 (0.58, 1.11)	0.80 (0.58, 1.11)	0.80 (0.45, 1.43)	0.81 (0.45, 1.43)	0.89 (0.55, 1.44)	0.88 (0.54, 1.44)	0.77 (0.38, 1.56)	0.76 (0.37, 1.53)
Adulthood SES								
Low	Ref	Ref	Ref	Ref	Ref	Ref	Ref	Ref
Middle	0.94 (0.71, 1.24)	0.94 (0.71, 1.24)	0.83 (0.53, 1.30)	0.85 (0.54, 1.33)	1.13 (0.74, 1.72)	1.15 (0.74, 1.77)	0.82 (0.44, 1.55)	0.82 (0.44, 1.53)
High	0.84 (0.62, 1.14)	0.84 (0.62, 1.13)	0.97 (0.60, 1.58)	1.00 (0.62, 1.63)	0.72 (0.43, 1.20)	0.74 (0.44, 1.24)	0.78 (0.42, 1.47)	0.79 (0.42, 1.48)
Social mobility								
Stable low SES	Ref	Ref	Ref	Ref	Ref	Ref	Ref	Ref
Downward mobility	0.88 (0.59, 1.32)	0.87 (0.58, 1.31)	0.91 (0.45, 1.84)	0.88 (0.43, 1.77)	0.80 (0.43, 1.49)	0.79 (0.42, 1.49)	1.51 (0.52, 4.37)	1.50 (0.52, 4.36)
Upward mobility	1.00 (0.70, 1.44)	1.00 (0.69, 1.44)	0.95 (0.41, 2.19)	0.93 (0.40, 2.16)	0.99 (0.59, 1.65)	1.02 (0.60, 1.72)	1.04 (0.52, 2.04)	1.04 (0.53, 2.05)
Stable middle or high SES	0.74 (0.51, 1.07)	0.73 (0.51, 1.06)	0.80 (0.41, 1.59)	0.80 (0.40, 1.59)	0.75 (0.44, 1.29)	0.77 (0.45, 1.32)	0.60 (0.27, 1.36)	0.60 (0.26, 1.34)

a Minimally adjusted model: adjusted for age, sex, and country.

b Fully adjusted model: additionally adjusted for marital status, smoking status, and daily alcohol consumption.

SES: socioeconomic status.

The results were more mixed when examining specific cancer types. There was less evidence for the protective effects of higher adulthood SES and upward mobility or stable middle or high SES in terms of deaths from lung cancer and colorectal cancer. However, in the fully adjusted model, compared to low adulthood SES, high adulthood SES was (borderline) significantly associated with 21% lower subhazard of death from lung cancer, given the fact that death from other cancers and other causes could also occur.

Two sets of sensitivity analyses were conducted. First, the associations of cancer mortality with social mobility in models with childhood SES measured by both parental education and household amenities at age 10 were stronger but generally similar to models with childhood SES measured by only household amenities at age 10 (Tables S12 and S13). Second, since health behaviours may act as mediators, rather than confounders, a mediation analysis was conducted in which a substantial proportion of the association between social mobility and cancer mortality was mediated by smoking (Fig. S4 and Table S14), suggesting that the adjusted models may underestimate the primary role of SES and social mobility.

### Gender disparities

The interaction term between social mobility and sex was tested to identify potential effect modification in the associations of social mobility with total, lung, and colorectal cancer mortality. There were no significant interactions with sex after adjusting for confounders in all associations. Results stratified by sex are presented in Tables S5–S9.

### Between- and within-country variation

The between-country variation in ASMRs was larger in upward mobility group and stable low SES group and the variation was smaller in downward mobility group (Table 3). ASMRs in participants with high adulthood SES and stable middle or high SES remained lower than other groups across countries, indicating a protective effect of high SES against overall cancer mortality. The within-country variation in total cancer mortality was the greatest in Czech Republic and the smallest in Poland, with Lithuania in the middle. Figure S3 presents the crude cancer mortality rates by SES and social mobility.

**Table 3.** Age-standardized cancer mortality rates per 1000 person-years by socioeconomic status and social mobility groups in Central and Eastern Europe

	Age-standardized cancer mortality rates per 1000 person-years											
	Total				Lung				Colorectal			
	All	CZ	PL	LT	All	CZ	PL	LT	All	CZ	PL	LT
Childhood SES												
Low	6.59	7.15	7.66	5.69	1.21	0.94	1.47	0.98	0.94	1.04	1.18	0.76
Middle	6.45	5.93	7.26	5.94	1.32	1.26	2.05	0.56	0.77	0.90	0.80	0.48
High	7.10	6.88	7.78	6.15	1.59	2.39	1.87	0.95	0.74	0.58	0.60	0.67
Adulthood SES												
Low	7.05	7.20	7.57	6.23	1.57	1.74	1.74	0.98	0.89	1.00	0.95	0.76
Middle	6.54	6.32	7.98	5.97	1.24	0.89	1.69	1.05	0.91	0.82	1.25	0.66
High	5.68	5.91	6.20	5.13	1.09	1.47	1.67	0.70	0.70	1.17	0.45	0.62
Social mobility												
Stable low SES	7.09	6.08	7.64	6.05	1.35	1.26	1.38	1.09	0.87	0.95	0.89	0.67
Downward mobility	7.43	7.41	8.01	7.30	1.79	1.89	2.19	0.64	1.05	0.96	1.22	1.13
Upward mobility	6.29	7.65	7.65	5.62	1.14	0.72	1.56	0.96	0.96	1.05	1.40	0.80
Stable middle or high SES	6.28	5.71	7.09	5.79	1.22	1.26	1.83	0.75	0.66	0.82	0.54	0.44
Total	6.50	6.65	7.43	5.62	1.31	1.37	1.69	0.87	0.86	0.97	0.97	0.70

CZ: Czech Republic, PL: Poland, LT: Lithuania, SES: socioeconomic status.

## Discussion

### Main findings of this study

In these three Central and Eastern European population-based cohorts, adulthood SES was inversely associated with deaths from total cancer and lung cancer in later life, while there was insufficient evidence of the role of childhood SES. Regarding social mobility, stable middle or high SES was associated with reduced total cancer mortality compared to stable low SES, while the association between upward social mobility and cancer mortality was attenuated after adjusting for health behaviours. We found no significant association between downward social mobility and cancer mortality. There were between-cohort variations in total, lung, and colorectal cancer mortality rates and their associations with social mobility.

### Comparison with existing literature

The evidence from western studies shows an inverse social gradient (by adulthood SES) in mortality from cancer [28–32]. For example, evidence from UK shows that lung cancer mortality is higher in manual than non-manual groups in men and women [31], and evidence from Belgium indicates that cancer mortality is higher in lower educated groups than the high educated group in men [32]. Our results from three cohorts in CEE found a similar gradient by adulthood SES. Given the fact that the participants of these cohorts spent the majority of their lives in socialist countries during the time of communist regimes pursuing (at least officially) egalitarian social and economic policies, one might have expected a lack, or less pronounced, social differences in health. In fact, previous studies found the opposite: that social differentials in all-cause mortality rates in Eastern Europe were, if anything, larger than in western countries [33], and similar findings seem to apply to cancer mortality [5].

Previous evidence from Europe indicates that cancer mortality trends are partly driven by levels and trends of cancer mortality in low education groups among middle-aged and older adults [5], and the current results show that rates of total cancer, lung cancer, and colorectal cancer are the highest in low adulthood SES group and that the between-country variation of ASMRs in low adulthood SES group is larger than in the high adulthood SES group. Although SES was measured differently in the current study compared with the previous European study [5], both studies showed the same trend for the role of adulthood SES than of childhood SES, which is consistent with previous findings from other European countries, such as Norway and Sweden [17, 19]. One exception is stomach cancer, which is more influenced by childhood SES [19, 34]. However, we did not investigate the association between SES and stomach cancer separately, and we could not compare with the previous results on the independent effect of childhood SES on stomach cancer.

Existing evidence on social mobility and cancer mortality is less extensive. The association between social mobility and cancer has been reported in Australia and Western and Northern Europe [18, 21, 22, 35–37]. Despite some inconsistencies among studies, stable low SES was associated with an increased risk of death from cancer in both men and women, compared with stable high SES (measured by father’s and one’s own occupation) [18, 21]. In France, downward intergenerational mobility was associated with an increased risk of death from smoking-related cancers in men and all cancers in women [18]. In Sweden, intragenerational upward mobility (assessed by occupation) was associated with a lower risk of death from cancer in men [22, 36], whereas the association between intragenerational downward mobility and cancer mortality was attenuated to non-significance after adjusting for sickness absence [22]. Using social mobility based on household income, stable low SES and downward mobility were associated with a higher risk of incident cancer compared to stable high SES in both sexes in Australia [35]. Regardless of the different measurements of social mobility, the current results from CEE are still consistent with these previous findings, with stable high SES was associated with a lower risk of death from cancer in both men and women compared to stable low SES.

In contrast to studies on childhood or adulthood SES (SES measured at a single period of life), social mobility accounts for a longer period. Its effect may accumulate and act throughout the life course [21]. In our study, we only considered risky health behaviours as mediators of childhood SES, but education, occupation, and household income in adulthood may modify risk factors. Therefore, intergenerational social mobility, which may lead to accumulation or attenuation of disadvantage (depending on the mobility direction), may provide additional information on health effects of SES on health.

In the conceptual framework of social determinants of health proposed by Marmot and Wilkinson [38], social structure influences well-being and health via material, psychosocial, and behavioural pathways. In this study, the proportion of current smokers was the highest in low adulthood SES group (Table S15) and smoking may partly mediate the association with cancer mortality, although we did not have detailed data on temporal sequence of events, consistent with findings from CEE and North Europe [11, 39]. Therefore, health behaviours might be a pathway linking SES or social mobility and later-life cancer mortality in CEE. International evidence suggests social gradients in cancer survival and late detection [6, 9, 40], which are important factors for cancer survival and, subsequently, for mortality. Unfortunately, we did not have information on cancer stage at diagnosis.

### Strengths and limitations

This research has several notable strengths. First, this is the first individual-based prospective multi-cohort study in CEE to investigate the association between social mobility and cancer. It provides novel evidence for the association between childhood and adulthood SES and social mobility and cancer mortality in this area. Second, the HAPIEE study has over 20 years of follow-up, which is an essential condition for observing death from chronic diseases such as cancer. Third, we used competing risk models to account for deaths from other causes. Unlike Cox proportional hazard models, which treat failures from competing causes as censored observations and therefore tend to be biased (overestimate the probability of failure), competing risk models provide more accurate estimates by accounting for competing events [26].

However, several limitations should be considered. First, the definition of childhood SES is inconsistent, due to the limitation in data availability. While childhood SES in Czech Republic was measured only by household amenities at age 10, in Poland and Lithuania it included parental education and household amenities at age 10. However, regardless of the differences in the strength of the relationship when using different measurements of childhood SES, direction of the association between social mobility and cancer mortality in the pooled sample remained the same in the main analysis and the sensitivity analyses. Second, parental education and household amenities were self-reported, and potential recall bias could not be excluded. Third, social mobility was based on only two time points, again due to data availability, and we were unable to estimate longer-term socioeconomic trajectories throughout the life course. Fourth, given the smaller sample size in each country and the fewer cases of lung or colorectal cancer compared to total cancer, statistical power of country- and sex-specific analyses, was limited. Finally, we were unable to separate the effects of incidence vs. survival on the resulting mortality risks, since two of the HAPIEE cohorts have not yet been linked with cancer registrations.

## Conclusions

Overall, this study found that adulthood SES but not childhood SES was associated with cancer mortality in later life in CEE countries. Compared to stable low SES, stable middle or high SES was associated with reduced risk of total cancer mortality, and the social patterning of cancer mortality was generally similar across the three countries. Cancer prevention and intervention programmes should target adults with socioeconomic disadvantages regardless of their childhood SES, since this group includes adults who have experienced downward social mobility.

## Supplementary Material

ckag124_Supplementary_Data

## Data Availability

The data underlying this article will be shared on reasonable request to the corresponding author. More information can be found at https://www.ucl.ac.uk/epidemiology-health-care/hapiee-study. Key points In these three Central and Eastern European cohorts (Czechia, Poland, Lithuania), high adulthood socioeconomic status (SES) was associated with lower total and lung cancer mortality in later life, compared with low adulthood SES. Stable middle or high SES was associated with lower total cancer mortality than stable low SES, given death from other causes could also occur. Effects of downward and upward social mobility on cancer mortality were less consistent, depending on cancer type and country.
