# Supplementary material for: Social mobility and cancer mortality in Central and Eastern Europe: a multicohort study
Source: Eur J Public Health. 2026 Jul 11;36(4):ckag124. doi: 10.1093/eurpub/ckag124 (PMC13356042; doi:10.1093/eurpub/ckag124)
Supplement: ckag124_Supplementary_Data [file ckag124_supplementary_data.zip › ejph-2025-12-om-1051-File002.docx]

**Manuscript Title: Social mobility and cancer mortality in Central and Eastern Europe: a multicohort study**

**Supplementary materials – tables**

**Table S1** Key characteristics of previous studies on socioeconomic status, social mobility and cancer

| (Reference number in the main text)  Study Title | Publication Year | Study Period | Study Area | Study Design | Study Population | Main exposure | Outcome |
| --- | --- | --- | --- | --- | --- | --- | --- |
| *Studies cited in Introduction* |  |  |  |  |  |  |  |
| (2) Cancer burden in Europe: a systematic analysis of the GLOBOCAN database | 2025 | Up to 2022 | 40 European countries | Population-based ecological study | General population | Country/region | Incidence and mortality of total cancer, prostate cancer, breast cancer, lung cancer |
| (3) Cancer survival in Europe 1999–2007 by country and age: results of EUROCARE-5—a population-based study | 2014 | 1999-2007 | 29 European countries | Population-based retrospective observational study | Adults diagnosed with cancer | Country/region | Cancer survival (5-year relative survival) |
| (4) Incidence and mortality of lung cancer: global trends and association with socioeconomic status | 2017 | Up to 2012 | Worldwide | Population-based ecological study | General population | Country-level socioeconomic indicators and calendar years | Incidence and mortality of lung cancer |
| (5) Socioeconomic inequalities in cancer mortality between and within countries in Europe: a population-based study | 2022 | 1990-2015 | 18 European countries | Population-based ecological study | Adults aged 40-79 years old | Education | Cancer mortality (all types) |
| (6) Association between socioeconomic position and lung cancer incidence in 16 countries: a prospective cohort consortium study | 2025 | Enrolled 1985-2010, median within-cohort  follow-up between 9.6 years  and 30 years | 16 countries in North America, Europe, Asia, and Australia | Prospective cohort  consortium study | Adults (mean age 58) | Education | Lung cancer incidence |
| (7) Mediating Role of Lifestyle Behaviors in the Association between Education and Cancer: Results from the European Prospective Investigation into Cancer and Nutrition | 2023 | Enrolled 1992-1999, median follow-up time 11 years | 10 European countries: France, Italy, Spain, UK, the Netherlands, Germany, Sweden, Denmark, Norway | Prospective cohort  study | Adults (mean age 50) | Education | Cancer incidence (overall and by sites) |
| (8) Lung, Breast and Colorectal Cancer Incidence by Socioeconomic Status in Spain: A Population-Based Multilevel Study | 2021 | Incident cases diagnosed for the period 2010–2013 | Spain (southern Europe) | Population-based ecological study | Males and females from nine Spanish provinces | Spanish Deprivation Index | Lung, breast and colorectal cancer incidence |
| (9) Socioeconomic inequalities in cancer incidence in Europe: a comprehensive review of population-based epidemiological studies | 2020 | Studies published between 2000-2019 | Europe | Review | Adults | SES | Cancer incidence |
| (10) The socioeconomic distribution of non-communicable diseases in Europe: findings from the European Social Survey (2014) special module on the social determinants of health | 2017 | 2014 European Social Survey | 20 European countries | Cross-sectional study | Respondents aged 25-75 | Education | 14 NCDs including cancer |
| (11) Socioeconomic inequalities in lung cancer mortality in 16 European populations | 2009 | 1990-2002 | 16 countries or regions, including  4 Eastern European and 2 Baltic countries | Population-based study | Adults aged 40-79 years old | Education | Lung cancer mortality |
| (12) The reversed social gradient: Higher breast cancer mortality in the higher educated compared to lower educated. A comparison of 11 European populations during the 1990s | 2007 | 1990s | 11 European  countries/regions | Population-based ecological study | Women aged 30-69 years at baseline | Education | Breast cancer mortality |
| (13) Childhood socioeconomic position and later-life mortality, morbidity and self-rated health: a linked study from the Historical Population Register of Norway and the Tromsø Study 1950–2022 | 2025 | 1994-2016 | Tromsø municipality, Norway | Prospective longitudinal study | Women and men aged 50 years and older | Father’s occupation in 1950 | All-cause mortality, chronic disease prevalence (including cancer) and self-rated health |
| (14) Childhood socioeconomic status, healthy lifestyle, and colon cancer risk in a cohort of U.S. women | 2022 | 1976-2016 | The USA | Cohort study | Female registered nurses aged 30-55 | Parents’ occupation at age 16 | Colon cancer incidence |
| (15) Effect of childhood socioeconomic conditions on cancer onset in later life: an ambidirectional cohort study | 2018 | 2004-2016 | 14 European countries: Austria, Belgium, Czech Republic, Denmark, France, Germany, Greece,  Ireland, Italy, the Netherlands, Poland,Spain, Sweden, Switzerland | Prospective longitudinal study | Men and women aged 50 years or older | Living conditions at age 10 | Cancer incidence (overall and by site) |
| (16) Childhood and adulthood socioeconomic position across 20 causes of death: a prospective cohort study of 800 000 Norwegian men and women | 2007 | 1990-2001 | Norway | Prospective cohort study | Men and women | Parental occupational class from 1960, household income in 1990 | Cause-specific mortality including cancer |
| (17) Childhood Socioeconomic Position and Cause-specific Mortality in Early Adulthood | 2007 | 1990-2001 | Norway | Cohort study | Men and women born in 1955-1965 | parents’ education, father’s occupation, household income | Cause-specific mortality including cancer, lung cancer, breast cancer |
| (18) Lifelong socioeconomic trajectory and premature mortality (35–65 years) in France: findings from the GAZEL Cohort Study | 2006 | 1990-2004 | France | Cohort study | Men (40-50 yrs) and women (35-50 yrs) employed by the French national gas and electricity company | Lifelong socioeconomic trajectories (father’s occupation, own occupation in young adulthood and in mid-life) | Premature (65 years) mortality (all-cause, smoking-related cancer, diseases of the circulatory system and external causes) |
| (19) Association of Childhood Socioeconomic Position with Cause-specific Mortality in a Prospective Record Linkage Study of 1,839,384 Individuals | 2006 | 1970-2001 | Sweden | Prospective record linkage study | Males and females born between 1944-1960 | Occupational social class of the head of the household at age 0-16, later-life occupation-based social class of the head of household | All-cause and cause-specific death (including cancer) |
| (20) Socioeconomic Position in Childhood and Early Adult Life and Risk of Mortality: A Prospective Study of the Mothers of the 1958 British Birth Cohort | 2005 | 1958-2003 | UK | Cohort study | British women aged 14 to 49 years | Father’s occupation when they left school, adult social class (husband’s occupation) | Cause-specific mortality including stomach, lung, breast cancer |
| (21) Social mobility and 21 year mortality in a cohort of Scottish men | 1998 | Screened in 1970s | Scotland | Cohort study | Men aged 35-64 years in 27 workplaces in the west of Scotland | Social mobility (father's occupation, their own first occupation, occupation at screening) | Cause-specific mortality including cancer mortality |
| (22) Sick leave absence and the relationship between intra-generational social mobility and mortality: health selection in Sweden | 2020 | 1996-2012 | Sweden | Swedish register data | Men and women who  had an occupation registered in 1996 and who were 65 years or younger | Social mobility based on occupational class | Cause-specific mortality including cancer |
| *Studies cited in Discussion* |  |  |  |  |  |  |  |
| (28) Inequalities in lung cancer mortality by the educational level in 10 European populations | 2004 | 1990s | England/Wales, Norway, Denmark, Finland, Belgium, Switzerland, Austria, Barcelona, Madrid, Turin | Population-based study | Adults aged 40-90 years | Education | Lung cancer mortality |
| (29) Socioeconomic inequalities in alcohol related cancer mortality among men: To what extent do they differ between Western European populations? | 2007 | 1990s | Western Europe | Population-based study | Men aged 30-74 years | Education | Alcohol-related cancer mortality |
| (30) Educational differences in cancer mortality among women and men: a gender pattern that differs across Europe | 2008 | 1990s | Western Europe | Population-based study | Women and men aged 30-74 years | Education | Cancer mortality (by sites) |
| (31) Social class differences in lung cancer mortality: risk factor explanations using two Scottish cohort studies | 2001 | Screened in 1970s | Scotland | Prospective cohort study | Men and women | Occupation (manual/non-manual) | Lung cancer mortality |
| (32) Evolution of educational inequalities in site-specific cancer mortality among Belgian men between the 1990s and 2000s using a “fundamental cause” perspective | 2017 | 1991-2001 | Belgium | Belgian censuses linked with register data on mortality | Men aged 50-79 | Education | Cancer mortality (by sites) |
| (33) Socioeconomic Inequalities in Health in 22 European Countries | 2008 | 1990-2004 | 22 European countries | Census-based mortality studies | Adults aged 30 to 74 years | Education, occupation | Cause-specific mortality including cancer mortality |
| (34) Socioeconomic position in childhood and cancer in adulthood: a rapid-review | 2016 | Studies published between 1996-2012 | UK, Norway, Sweden,  Finland, Denmark, the Netherlands, France, the USA | Review | Adults | Childhood SES | Cancer incidence and mortality in adulthood |
| (35) Determinants and health outcomes of trajectories of social mobility in Australia | 2023 | 2001-2020 | Australia | Prospective cohort study | Men and women residing in Australia | Social mobility based on household income | Onset of physical and mental health outcomes including cancer |
| (36) Intragenerational social mobility and cause-specific premature mortality | 2019 | 1996-2012 | Sweden | Prospective, population-based register data | Men and  Women working in 1996 up to age 65 | Social mobility based on occupational class | Cause-specific mortality including cancer |
| (37) Socioeconomic status, social mobility and cancer occurrence during working life: a case-control study among French electricity and gas workers | 1999 | 1988-1992 | France | Case-control study | French electricity and gas workers | SES and social mobility based on employee category and French SES classification system | Cancer incidence (by sites) |
| (39) Educational inequalities in cancer mortality differ greatly between countries around the Baltic Sea | 2008 | 1990s-2000s | Poland, Lithuania, Estonia, Finland, and Sweden | Follow-up or unlinked cross-sectional study | Adults aged 35-79 years | Education | Cancer mortality (overall and by sites) |
| (40) Social inequality in incidence of and survival from cancer in a population-based study in Denmark, 1994–2003: Summary of findings | 2008 | 1994-2006 | Denmark | Population register-based study | Danish residents born in 1925–1973 and aged  ≥30 years | Social position | Cancer incidence and survival (overall and by sites) |

**Table S2** Cross-tabulation of two measures of childhood SES in all countries and in Czech Republic (parental education was only available on subsample in the Czech Republic)

|  | **Childhood SES^b^**  ***n (row %)*** | | | | | |
| --- | --- | --- | --- | --- | --- | --- |
|  | **All countries** | | | **Czech Republic** | | |
| **Childhood SES^a^** | Low | Middle | High | Low | Middle | High |
| Low | 8,138  (93.51) | 554  (6.37) | 11  (0.13) | 241  (29.90) | 554  (68.73) | 11  (1.36) |
| Middle | 312  (5.59) | 5,083  (91.04) | 188  (3.37) | 312  (15.48) | 1,516  (75.20) | 188  (9.33) |
| High | 4  (0.07) | 105  (1.89) | 5460  (98.04) | 4  (0.25) | 105  (6.69) | 1,460  (93.05) |
| ***Total*** | 8,454  (42.58) | 5,742  (28.92) | 5,659  (28.50) | 557  (12.69) | 2,175  (49.53) | 1,659  (37.78) |

a: Childhood SES measured by both parental education and household amenities at age 10 in all three countries.

b: Childhood SES measured by only household amenities at age 10 in Czech Republic, and measured by both parental education and household amenities in Poland and Lithuania.

Notes: Due to the large number of missing data on parental education in Czech Republic, childhood SES 1, measured by both parental education and household amenities at age 10, is only available for 4,391 Czech participant. Childhood SES 2, measured by only household amenities at age 10, is available for all 7,545 Czech participants. This table includes 19,855 participants (4,391 of them from Czech Republic) with complete data on both measures of childhood SES measurements.

**Table S3** Baseline sample characteristics according to SES and social mobility in Central and Eastern European countries

| **Sample characteristics** | | **Social mobility n (col %)** | | | | **P-values^a^** |
| --- | --- | --- | --- | --- | --- | --- |
|  |  | Stable middle or high SES | Upward mobility | Downward mobility | Stable low SES |  |
| Age (year) | Mean (SD) | 57.16 (7.15) | 61.64 (6.79) | 56.47 (7.06) | 60.87 (6.93) | <0.001 |
| Sex | Male  Female | 4680 (50.52)  4583 (49.48) | 3006 (52.21)  2752 (47.79) | 2020 (41.17)  2887 (58.83) | 1193 (38.72)  1888 (61.28) | <0.001 |
| Marital status | Single  Married/cohabiting  Divorced/separated/widowed | 372 (4.02)  7467 (80.61)  1424 (15.37) | 177 (3.07)  4445 (77.20)  1136 (19.73) | 236 (4.81)  3313 (67.52)  1358 (27.67) | 134 (4.35)  2015 (65.40)  932 (30.25) | <0.001 |
| Smoking status | Never smoker  Former smoker  Current smoker | 4232 (45.69)  2589 (27.95)  2442 (26.36) | 3245 (56.36)  1450 (25.18)  1063 (18.46) | 1832 (37.33)  1252 (25.51)  1823 (37.15) | 1550 (50.31)  730 (23.69)  801 (26.00) | <0.001 |
| Daily alcohol consumption (g/day) | Mean (SD) | 202.91 (412.01) | 104.30 (294.14) | 196.95 (391.12) | 98.38 (271.73) | <0.001 |
|  | | **Childhood SES n (col %)** | | |  |  |
|  |  | High | Middle | Low |  |  |
| Age (year) | Mean (SD) | 54.72 (6.68) | 59.04 (6.89) | 61.37 (6.85) | - | <0.001 |
| Sex | Male  Female | 3287 (47.26)  3668 (52.74) | 3413 (47.30)  3802 (52.70) | 4199 (47.51)  4640 (52.49) |  | 0.95 |
| Marital status | Single  Married/cohabiting  Divorced/separated/widowed | 355 (5.10)  5265 (75.70)  1335 (19.19) | 253 (3.51)  5515 (76.44)  1447 (20.06) | 311 (3.52)  6460 (73.09)  2068 (23.40) |  | <0.001 |
| Smoking status | Never smoker  Former smoker  Current smoker | 2890 (41.55)  1780 (25.59)  2285 (32.85) | 3174 (43.99)  2061 (28.57)  1980 (27.44) | 4795 (54.25)  2180 (24.66)  1864 (21.09) |  | <0.001 |
| Daily alcohol consumption (g/day) | Mean (SD) | 204.38 (394.07) | 197.45 (415.07) | 102.24 (286.53) |  | <0.001 |
|  | | **Adulthood SES n (col %)** | | |  |  |
|  |  | High | Middle | Low |  |  |
| Age (year) | Mean (SD) | 57.22 (7.09) | 60.67 (7.19) | 58.17 (7.33) | - | <0.001 |
| Sex | Male  Female | 4324 (55.26)  3501 (44.74) | 3362 (46.72)  3834 (53.28) | 3213 (40.22)  4775 (59.78) |  | <0.001 |
| Marital status | Single  Married/cohabiting  Divorced/separated/widowed | 251 (3.21)  6486 (82.89)  1088 (13.90) | 298 (4.14)  5426 (75.40)  1472 (20.46) | 370 (4.63)  5328 (66.70)  2290 (28.67) |  | <0.001 |
| Smoking status | Never smoker  Former smoker  Current smoker | 3788 (48.41)  2141 (27.36)  1896 (24.23) | 3689 (51.26)  1898 (26.38)  1609 (22.36) | 3382 (42.34)  1982 (24.81)  2624 (32.85) |  | <0.001 |
| Daily alcohol consumption (g/day) | Mean (SD) | 180.51 (387.10) | 148.37 (359.25) | 158.93 (353.19) |  | <0.001 |

a: P-values from chi-squared test or Kruskal-Wallis test for the differences between SES groups.

SD: standard deviation.

**Table S4** Baseline sample characteristics according to causes of death in Central and Eastern European countries

| **Sample characteristics** | | **Alive**  **n=15774**  n (col%) | **Other/ unknown causes**  **n=4852**  n (col %) | **Total cancer**  **n=2383** | | **Lung cancer**  **n=497** | | **Colorectal cancer**  **n=297** | |
| --- | --- | --- | --- | --- | --- | --- | --- | --- | --- |
|  |  |  |  | n of death (col %) | p-values^a^ | n of death  (col %) | p-values^a^ | n of death  (col %) | p-values^a^ |
| Age (year) | Mean (SD) | 56.84 (6.97) | 62.93 (6.56) | 61.69 (6.61) | <0.001 | 61.01 (6.72) | <0.001 | 62.46 (6.67) | <0.001 |
| Sex | Male  Female | 6585 (41.75)  9189 (58.25) | 2911 (60.00)  1941 (40.00) | 1403 (58.88)  980 (41.12) | <0.001 | 345 (69.42)  152 (30.58) | <0.001 | 192 (64.65)  105 (35.35) | <0.001 |
| Marital status | Single  Married/cohabiting  Divorced/separated/widowed | 620 (3.93)  12022 (76.21)  3132 (19.86) | 214 (4.41)  3430 (70.69)  1208 (24.90) | 85 (3.57)  1788 (75.03)  510 (21.40) | 0.51 | 14 (2.82)  395 (79.48)  88 (17.71) | 0.05 | 7 (2.36)  232 (78.11)  58 (19.53) | 0.25 |
| Smoking status | Never smoker  Former smoker  Current smoker | 8017 (50.82)  3917 (24.83)  3840 (24.34) | 1994 (41.10)  1424 (29.35)  1434 (29.55) | 848 (35.59)  680 (28.54)  855 (35.88) | <0.001 | 61 (12.27)  126 (25.35)  310 (62.37) | <0.001 | 113 (38.05)  114 (38.38)  70 (23.57) | <0.001 |
| Daily alcohol consumption (g/day) | Mean (SD) | 149.95 (350.64) | 190.85 (398.83) | 192.39 (400.48) | <0.001 | 232.21 (453.95) | <0.001 | 236.73 (391.21) | <0.001 |

a: P-values from chi-squared test, chi-squared test for trend, or Kruskal-Wallis test for the differences between participants who died from cancer (any cancer, lung cancer, or colorectal cancer) and those who remained alive or died from other/unknown causes.

SD: standard deviation.

**Table S5** Baseline sample characteristics according to country of residence in males and females, HAPIEE

| **Sample characteristics** | | **N (col %)** | | | | **N of death from cancer** | **Cancer mortality rate per 1000 person-years** |
| --- | --- | --- | --- | --- | --- | --- | --- |
|  |  | Total  n=23009 | Czech Republic  n=7545 (row%=32.79) | Poland  n=9252 (row%=40.21) | Lithuania  n=6212 (row%=27.00) |  |  |
| **Males** | | 10899 (47.37) | 3572 (47.34) | 4514 (48.79) | 2813 (45.28) | 1403 | 8.12 |
| **Age (year)** | Mean (SD) | 58.91 (7.33) | 58.49 (7.22) | 57.90 (6.95) | 61.08 (7.60) | 1403 | 8.12 |
| **Marital status** | Single  Married/cohabiting  Divorced/separated/widowed | 320 (2.94)  9350 (85.79)  1229 (11.28) | 101 (2.83)  3015 (84.41)  456 (12.77) | 166 (3.68)  3929 (87.04)  419 (9.28) | 53 (1.88)  2406 (85.53)  354 (12.58) | 34  1188  181 | 7.12  7.93  9.97 |
| **Smoking status** | Never smoker  Former smoker  Current smoker | 3486 (31.98)  3904 (35.82)  3509 (32.20) | 1140 (31.91)  1383 (38.72)  1049 (29.37) | 1255 (27.80)  1629 (36.09)  1630 (36.11) | 1091 (38.78)  892 (31.71)  830 (29.51) | 308  518  577 | 5.25  8.47  10.89 |
| **Daily alcohol consumption (g/day)** | Mean (SD) | 285.49 (459.59) | 505.03 (568.23) | 209.37 (406.06) | 128.87 (221.11) | 1403 | 8.12 |
| **Household amenities at age 10** | High (5 or 6 items)  Medium (3-4 items)  Low (0-2 items) | 3159 (28.98)  4428 (40.63)  3312 (30.39) | 1372 (38.41)  1711 (47.90)  489 (13.69) | 1448 (32.08)  1253 (27.76)  1813 (40.16) | 339 (12.05)  1464 (52.04)  1010 (35.90) | 315  594  494 | 5.80  8.50  10.16 |
| **Father’s education^a^** | University/college  Secondary/vocational  Incomplete secondary/primary  Incomplete primary or lower | 1009 (13.77)  2105 (28.73)  3424 (46.73)  789 (10.77) | - | 511 (11.32)  1714 (37.97)  1900 (42.09)  389 (8.62) | 498 (17.70)  391 (13.90)  1524 (54.18)  400 (14.22) | 110  234  452  121 | 7.08  7.10  8.88  11.22 |
| **Mother’s education^a^** | University/college  Secondary/vocational  Incomplete secondary/primary  Incomplete primary or lower | 525 (7.17)  1951 (26.63)  3890 (53.09)  961 (13.12) | - | 182 (4.03)  1548 (34.29)  2320 (51.40)  464 (10.28) | 343 (12.19)  403 (14.33)  1570 (55.81)  497 (17.67) | 54  209  504  150 | 6.57  6.78  8.69  11.45 |
| **Childhood SES** | High  Middle  Low | 3287 (30.16)  3413 (31.31)  4199 (38.53) | 1372 (38.41)  1711 (47.90)  489 (13.69) | 1295 (28.69)  1248 (27.65)  1971 (43.66) | 620 (22.04)  454 (16.14)  1739 (61.82) | 342  448  613 | 6.11  8.08  9.98 |
| **Employment status** | Employed/self-employed  Other  Not employed pensioner/unemployed | 5180 (47.53)  1110 (10.18)  4609 (42.29) | 1783 (49.92)  316 (8.85)  1473 (41.24) | 1848 (40.94)  362 (8.02)  2304 (51.04) | 1549 (55.07)  432 (15.36)  832 (29.58) | 441  159  803 | 4.87  9.64  12.21 |
| **Education** | University/college  Secondary/vocational  Primary or lower | 3582 (32.87)  6379 (58.53)  938 (8.61) | 698 (19.54)  2678 (74.97)  196 (5.49) | 1358 (30.08)  2752 (60.97)  404 (8.95) | 1526 (54.25)  949 (33.74)  338 (12.02) | 371  870  162 | 6.41  8.51  12.77 |
| **Number of households amenities^b^** | High (9 to 12 items)  Medium (5 to 8 items)  Low (less than 5 items) | 2761 (25.33)  6504 (59.68)  1634 (14.99) | 1013 (28.36)  2071 (57.98)  488 (13.66) | 1000 (22.15)  2811 (62.27)  703 (15.57) | 748 (26.59)  1622 (57.66)  443 (15.75) | 276  839  288 | 5.85  8.12  12.92 |
| **Absolute material deprivation** | Low  Medium  High | 1107 (10.16)  3237 (29.70)  6555 (60.14) | 311 (8.71)  1318 (36.90)  1943 (54.40) | 676 (14.98)  1420 (31.46)  2418 (53.57) | 120 (4.27)  499 (17.74)  2194 (78.00) | 160  421  822 | 9.77  8.06  7.89 |
| **Adulthood SES** | High  Middle  Low | 4324 (39.67)  3362 (30.85)  3213 (29.48) | 1267 (35.47)  1067 (29.87)  1238 (34.66) | 1501 (33.25)  1386 (30.70)  1627 (36.04) | 1556 (55.31)  909 (32.31)  348 (12.37) | 448  480  475 | 6.20  9.40  9.59 |
| **Social mobility** | Stable middle or high SES  Upward mobility  Downward mobility  Stable low SES | 4680 (42.94)  3006 (27.58)  2020 (18.53)  1193 (10.95) | 2045 (57.25)  289 (8.09)  1038 (29.06)  200 (5.60) | 1669 (36.97)  1218 (26.98)  874 (19.36)  753 (16.68) | 966 (34.34)  1499 (53.29)  108 (3.84)  240 (8.53) | 515  413  275  200 | 6.53  9.29  8.44  11.81 |
| **Status^c^** | Alive  Death from lung cancer  Death from colorectal cancer  Death from other cancers  Death from other causes  Death from unknown causes | 6585 (60.42)  345 (3.17)  192 (1.76)  866 (7.95)  2820 (25.87)  91 (0.83) | 2023 (56.63)  113 (3.16)  75 (2.10)  298 (8.34)  1035 (28.98)  28 (0.78) | 2905 (64.36)  159 (3.52)  72 (1.60)  339 (7.51)  981 (21.73)  58 (1.28) | 1657 (58.91)  73 (2.60)  45 (1.60)  229 (8.14)  804 (28.58)  5 (0.18) | - | - |
| **Females** |  | 12110 (52.63) | 3973 (52.66) | 4738 (51.21) | 3399 (54.72) | 980 | 4.65 |
| **Age (year)** | Mean (SD) | 58.37 (7.36) | 57.61 (7.09) | 57.31 (6.97) | 60.74 (7.64) | 980 | 4.65 |
| **Marital status** | Single  Married/cohabiting  Divorced/separated/widowed | 599 (4.95)  7890 (65.15)  3621 (29.90) | 93 (2.34)  2739 (68.94)  1141 (28.72) | 317 (6.69)  3174 (66.99)  1247 (26.32) | 189 (5.56)  1977 (58.16)  1233 (36.28) | 51  600  329 | 5.09  4.30  5.40 |
| **Smoking status** | Never smoker  Former smoker  Current smoker | 7373 (60.88)  2117 (17.48)  2620 (21.64) | 2151 (54.14)  874 (22.00)  948 (23.86) | 2389 (50.42)  997 (21.04)  1352 (28.54) | 2833 (83.35)  246 (7.24)  320 (9.41) | 540  162  278 | 4.23  4.30  6.13 |
| **Daily alcohol consumption (g/day)** | Mean (SD) | 52.69 (200.87) | 91.46 (202.31) | 40.54 (150.39) | 24.32 (248.28) | 980 | 4.65 |
| **Household amenities at age 10** | High (5 or 6 items)  Medium (3-4 items)  Low (0-2 items) | 3626 (29.94)  5131 (42.37)  3353 (27.69) | 1583 (39.84)  1937 (48.75)  453 (11.40) | 1645 (34.72)  1369 (28.89)  1724 (36.39) | 398 (11.71)  1825 (53.69)  1176 (34.60) | 239  432  309 | 3.59  4.84  5.64 |
| **Father’s education^a^** | University/college  Secondary/vocational  Incomplete secondary/primary  Incomplete primary or lower | 1032 (12.68)  2318 (28.49)  3899 (47.92)  888 (10.91) | - | 519 (10.95)  1833 (38.69)  1995 (42.11)  391 (8.25) | 513 (15.09)  485 (14.27)  1904 (56.02)  497 (14.62) | 81  170  306  66 | 4.71  4.38  4.79  4.72 |
| **Mother’s education^a^** | University/college  Secondary/vocational  Incomplete secondary/primary  Incomplete primary or lower | 603 (7.41)  2020 (24.82)  4393 (53.99)  1121 (13.78) | - | 219 (4.62)  1579 (33.33)  2476 (52.26)  464 (9.79) | 384 (11.30)  441 (12.97)  1917 (56.40)  657 (19.33) | 38  154  340  91 | 3.75  4.53  4.71  5.18 |
| **Childhood SES** | High  Middle  Low | 3668 (30.29)  3802 (31.40)  4640 (38.32) | 1583 (39.84)  1937 (48.75)  453 (11.40) | 1420 (29.97)  1310 (27.65)  2008 (42.38) | 665 (19.56)  555 (16.33)  2179 (64.11) | 266  319  395 | 3.98  4.70  5.21 |
| **Employment status** | Employed/self-employed  Other  Not employed pensioner/unemployed | 4725 (39.02)  1235 (10.20)  6150 (50.78) | 1637 (41.20)  339 (8.53)  1997 (50.26) | 1605 (33.88)  407 (8.59)  2726 (57.53) | 1483 (43.63)  489 (14.39)  1427 (41.98) | 207  108  665 | 2.37  5.11  6.52 |
| **Education** | University/college  Secondary/vocational  Primary or lower | 3814 (31.49)  6619 (54.66)  1677 (13.85) | 437 (11.00)  2855 (71.86)  681 (17.14) | 1292 (27.27)  2817 (59.46)  629 (13.28) | 2085 (61.34)  947 (27.86)  367 (10.80) | 248  558  174 | 3.80  4.73  6.33 |
| **Number of households amenities^b^** | High (9 to 12 items)  Medium (5 to 8 items)  Low (less than 5 items) | 2178 (17.99)  7231 (59.71)  2701 (22.30) | 827 (20.82)  2400 (60.41)  746 (18.78) | 732 (15.45)  2857 (60.30)  1149 (24.25) | 619 (18.21)  1974 (58.08)  806 (23.71) | 141  551  288 | 3.56  4.33  6.59 |
| **Absolute material deprivation** | Low  Medium  High | 1968 (16.25)  4073 (33.63)  6069 (50.12) | 533 (13.42)  1599 (40.25)  1841 (46.34) | 1076 (22.71)  1620 (34.19)  2042 (43.10) | 359 (10.56)  854 (25.13)  2186 (64.31) | 173  322  485 | 5.25  4.46  4.60 |
| **Adulthood SES** | High  Middle  Low | 3501 (28.91)  3834 (31.66)  4775 (39.43) | 951 (23.94)  1247 (31.39)  1775 (44.68) | 1103 (23.28)  1421 (29.99)  2214 (46.73) | 1447 (42.57)  1166 (34.30)  786 (23.12) | 220  344  416 | 3.52  5.25  5.04 |
| **Social mobility** | Stable middle or high SES  Upward mobility  Downward mobility  Stable low SES | 4583 (37.84)  2752 (22.73)  2887 (23.84)  1888 (15.59) | 1988 (50.04)  210 (5.29)  1532 (38.56)  243 (6.12) | 1574 (33.22)  950 (20.05)  1156 (24.40)  1058 (22.33) | 1021 (30.04)  1592 (46.84)  199 (5.85)  587 (17.27) | 340  224  245  171 | 4.10  4.96  4.72  5.56 |
| **Status^c^** | Alive  Death from lung cancer  Death from colorectal cancer  Death from other cancers  Death from other causes  Death from unknown causes | 9189 (75.88)  152 (1.26)  105 (0.87)  723 (5.97)  1875 (15.48)  66 (0.55) | 2920 (73.50)  64 (1.61)  37 (0.93)  256 (6.44)  679 (17.09)  17 (0.43) | 3705 (78.20)  69 (1.46)  38 (0.80)  272 (5.74)  607 (12.81)  47 (0.99) | 2564 (75.43)  19 (0.56)  30 (0.88)  195 (5.74)  589 (17.33)  2 (0.06) | - | - |

a: Total number of males and females with parental education is 15464.

b: Czech Republic and Poland:12 items; Lithuania:10 items.

c: Death from cancer in all countries in males and females: n=2383 (10.36%); Czech Republic: n=843 (11.17%); Poland: n=949 (10.26%); Lithuania: n=591 (9.51%).

SD: standard deviation; SES: socioeconomic status.

Percentages may not total 100% due to rounding.

**Table S6** Associations of childhood SES, adulthood SES, and social mobility with cancer mortality in Central and Eastern Europe in males: subdistribution hazard ratios and 95% confidence intervals

|  | **Subdistribution hazard ratios (95% confidence intervals)** | | | | | | | |
| --- | --- | --- | --- | --- | --- | --- | --- | --- |
|  | **All countries**  n=10899 | | **Czech Republic**  n=3572 | | **Poland**  n=4514 | | **Lithuania**  n=2813 | |
|  | Minimally adjusted^a^ | Fully adjusted^b^ | Age adjusted | Fully adjusted^b^ | Age adjusted | Fully adjusted^b^ | Age adjusted | Fully adjusted^b^ |
| ***(A) Total cancer*** |  | |  | |  | |  | |
| **Childhood SES**  Low  Middle  High | Ref  0.94 (0.82,1.08)  1.03 (0.89,1.19) | Ref  0.91 (0.80,1.04)  1.00 (0.87,1.17) | Ref  1.02 (0.79,1.31)  1.17 (0.87,1.58) | Ref  0.99 (0.77,1.28)  1.16 (0.86,1.57) | Ref  0.96 (0.78,1.17)  1.07 (0.86,1.32) | Ref  0.91 (0.74,1.11)  1.01 (0.81,1.26) | Ref  0.85 (0.63,1.16)  0.87 (0.64,1.19) | Ref  0.83 (0.61,1.13)  0.87 (0.64,1.19) |
| **Adulthood SES**  Low  Middle  High | Ref  0.81 (0.72,0.93)  0.73 (0.64,0.84) | Ref  0.88 (0.77,1.01)  0.82 (0.71,0.94) | Ref  0.84 (0.68,1.04)  0.76 (0.61,0.95) | Ref  0.90 (0.73,1.12)  0.83 (0.66,1.04) | Ref  0.79 (0.66,0.96)  0.71 (0.57,0.87) | Ref  0.89 (0.73,1.08)  0.81 (0.65,1.00) | Ref  0.79 (0.58,1.09)  0.71 (0.52,0.97) | Ref  0.83 (0.60,1.15)  0.77 (0.56,1.06) |
| **Social mobility**  Stable low SES  Downward mobility  Upward mobility  Stable middle or high SES | Ref  1.01 (0.84,1.23)  0.79 (0.66,0.94)  0.77 (0.65,0.92) | Ref  0.96 (0.80,1.17)  0.86 (0.72,1.02)  0.82 (0.69,0.97) | Ref  1.27 (0.86,1.87)  1.02 (0.65,1.59)  0.96 (0.66,1.40) | Ref  1.20 (0.82,1.77)  1.06 (0.68,1.66)  1.00 (0.69,1.45) | Ref  0.97 (0.74,1.27)  0.72 (0.57,0.91)  0.76 (0.60,0.97) | Ref  0.89 (0.68,1.17)  0.80 (0.63,1.02)  0.81 (0.64,1.03) | Ref  0.94 (0.52,1.70)  0.77 (0.55,1.08)  0.66 (0.45,0.97) | Ref  0.89 (0.49,1.62)  0.81 (0.57,1.15)  0.69 (0.47,1.02) |
| ***(B) Lung cancer*** |  | |  | |  | |  | |
| **Childhood SES**  Low  Middle  High | Ref  1.00 (0.76,1.31)  1.18 (0.89,1.58) | Ref  0.91 (0.69,1.19)  1.11 (0.83,1.47) | Ref  0.96 (0.56,1.65)  1.42 (0.75,2.70) | Ref  0.88 (0.51,1.52)  1.36 (0.72,2.57) | Ref  1.24 (0.85,1.81)  1.25 (0.84,1.87) | Ref  1.11 (0.77,1.62)  1.13 (0.76,1.69) | Ref  0.71 (0.35,1.44)  0.80 (0.41,1.56) | Ref  0.67 (0.33,1.36)  0.80 (0.41,1.55) |
| **Adulthood SES**  Low  Middle  High | Ref  0.76 (0.59,0.99)  0.64 (0.49,0.84) | Ref  0.90 (0.69,1.17)  0.80 (0.60,1.05) | Ref  0.54 (0.34,0.88)  0.80 (0.53,1.23) | Ref  0.63 (0.39,1.04)  0.97 (0.63,1.49) | Ref  0.87 (0.61,1.23)  0.62 (0.41,0.93) | Ref  1.03 (0.72,1.47)  0.75 (0.49,1.14) | Ref  0.80 (0.42,1.50)  0.47 (0.25,0.90) | Ref  0.95 (0.50,1.81)  0.67 (0.34,1.33) |
| **Social mobility**  Stable low SES  Downward mobility  Upward mobility  Stable middle or high SES | Ref  1.12 (0.77,1.62)  0.71 (0.50,1.01)  0.77 (0.55,1.09) | Ref  0.99 (0.69,1.44)  0.85 (0.59,1.21)  0.85 (0.60,1.19) | Ref  1.06 (0.51,2.23)  0.64 (0.25,1.62)  0.72 (0.35,1.48) | Ref  0.93 (0.45,1.96)  0.71 (0.27,1.83)  0.76 (0.37,1.57) | Ref  1.27 (0.78,2.07)  0.73 (0.46,1.17)  0.94 (0.59,1.47) | Ref  1.09 (0.66,1.78)  0.86 (0.53,1.38)  1.00 (0.64,1.56) | Ref  0.67 (0.19,2.40)  0.59 (0.30,1.15)  0.47 (0.22,1.00) | Ref  0.60 (0.16,2.24)  0.76 (0.38,1.53)  0.59 (0.26,1.32) |
| ***(C) Colorectal cancer*** |  | |  | |  | |  | |
| **Childhood SES**  Low  Middle  High | Ref  0.82 (0.58,1.16)  1.03 (0.70,1.52) | Ref  0.82 (0.58,1.16)  1.05 (0.71,1.54) | Ref  1.02 (0.56,1.84)  1.05 (0.54,2.03) | Ref  1.03 (0.57,1.85)  1.07 (0.55,2.07) | Ref  0.66 (0.36,1.24)  1.02 (0.58,1.82) | Ref  0.65 (0.34,1.21)  1.00 (0.56,1.80) | Ref  0.55 (0.20,1.55)  1.23 (0.55,2.74) | Ref  0.54 (0.19,1.50)  1.20 (0.54,2.68) |
| **Adulthood SES**  Low  Middle  High | Ref  0.73 (0.52,1.04)  0.69 (0.48,1.00) | Ref  0.74 (0.52,1.05)  0.70 (0.48,1.01) | Ref  0.64 (0.37,1.11)  0.73 (0.42,1.28) | Ref  0.65 (0.37,1.14)  0.76 (0.43,1.34) | Ref  0.98 (0.57,1.69)  0.75 (0.42,1.36) | Ref  1.02 (0.58,1.79)  0.78 (0.43,1.42) | Ref  0.52 (0.24,1.14)  0.54 (0.25,1.16) | Ref  0.53 (0.25,1.15)  0.53 (0.25,1.15) |
| **Social mobility**  Stable low SES  Downward mobility  Upward mobility  Stable middle or high SES | Ref  1.00 (0.61,1.66)  0.78 (0.49,1.23)  0.68 (0.43,1.07) | Ref  1.00 (0.60,1.66)  0.78 (0.49,1.22)  0.68 (0.44,1.08) | Ref  1.05 (0.46,2.40)  0.68 (0.25,1.88)  0.72 (0.32,1.62) | Ref  1.00 (0.43,2.30)  0.65 (0.23,1.80)  0.71 (0.31,1.61) | Ref  0.77 (0.34,1.73)  0.84 (0.44,1.59)  0.73 (0.38,1.39) | Ref  0.75 (0.33,1.71)  0.88 (0.46,1.68)  0.74 (0.39,1.42) | Ref  1.95 (0.55,6.92)  0.73 (0.30,1.76)  0.50 (0.17,1.43) | Ref  1.99 (0.55,7.20)  0.74 (0.31,1.79)  0.49 (0.17,1.41) |

a: Minimally adjusted model: adjusted for age and country.

b: Fully adjusted model: additionally adjusted for marital status, smoking status, and daily alcohol consumption.

SES: socioeconomic status.

**Table S7** Associations of childhood SES, adulthood SES, and social mobility with cancer mortality in Central and Eastern Europe in females: subdistribution hazard ratios and 95% confidence intervals

|  | **Subdistribution hazard ratios (95% confidence intervals)** | | | | | | | |
| --- | --- | --- | --- | --- | --- | --- | --- | --- |
|  | **All countries**  n=12110 | | **Czech Republic**  n= 3973 | | **Poland**  n= 4738 | | **Lithuania**  n= 3399 | |
|  | Minimally adjusted^a^ | Fully adjusted^b^ | Age adjusted | Fully adjusted^b^ | Age adjusted | Fully adjusted^b^ | Age adjusted | Fully adjusted^b^ |
| ***(A) Total cancer*** |  | |  | |  | |  | |
| **Childhood SES**  Low  Middle  High | Ref  0.98 (0.82,1.16)  1.12 (0.93,1.34) | Ref  0.95 (0.80,1.13)  1.08 (0.90,1.29) | Ref  0.79 (0.59,1.06)  1.06 (0.76,1.48) | Ref  0.79 (0.58,1.06)  1.05 (0.75,1.46) | Ref  0.98 (0.77,1.25)  0.97 (0.74,1.27) | Ref  0.95 (0.74,1.21)  0.94 (0.72,1.22) | Ref  1.33 (0.94,1.88)  1.37 (0.97,1.94) | Ref  1.31 (0.92,1.85)  1.34 (0.95,1.91) |
| **Adulthood SES**  Low  Middle  High | Ref  0.97 (0.84,1.12)  0.86 (0.73,1.02) | Ref  0.99 (0.86,1.15)  0.90 (0.76,1.06) | Ref  0.89 (0.71,1.13)  0.96 (0.72,1.27) | Ref  0.92 (0.73,1.17)  1.00 (0.75,1.33) | Ref  0.98 (0.78,1.23)  0.82 (0.62,1.08) | Ref  1.00 (0.80,1.26)  0.86 (0.65,1.13) | Ref  1.06 (0.77,1.45)  0.84 (0.60,1.18) | Ref  1.07 (0.77,1.47)  0.86 (0.61,1.20) |
| **Social mobility**  Stable low SES  Downward mobility  Upward mobility  Stable middle or high SES | Ref  1.09 (0.88,1.34)  0.96 (0.79,1.18)  0.97 (0.80,1.18) | Ref  1.04 (0.84,1.28)  0.98 (0.80,1.21)  0.97 (0.80,1.18) | Ref  0.99 (0.66,1.48)  1.18 (0.71,1.95)  0.87 (0.59,1.29) | Ref  0.95 (0.64,1.42)  1.17 (0.70,1.94)  0.88 (0.59,1.30) | Ref  1.03 (0.76,1.39)  0.96 (0.71,1.29)  0.91 (0.68,1.20) | Ref  0.98 (0.73,1.32)  0.98 (0.73,1.32)  0.90 (0.68,1.20) | Ref  1.54 (0.89,2.68)  0.97 (0.68,1.37)  1.27 (0.86,1.88) | Ref  1.50 (0.86,2.60)  0.98 (0.69,1.39)  1.26 (0.85,1.88) |
| ***(B) Lung cancer*** |  | |  | |  | |  | |
| **Childhood SES**  Low  Middle  High | Ref  1.23 (0.82,1.86)  1.52 (0.96,2.41) | Ref  1.12 (0.74,1.67)  1.34 (0.85,2.10) | Ref  1.61 (0.67,3.86)  2.10 (0.83,5.32) | Ref  1.64 (0.68,3.96)  2.09 (0.82,5.35) | Ref  1.36 (0.77,2.41)  1.29 (0.67,2.52) | Ref  1.18 (0.67,2.06)  1.10 (0.58,2.06) | Ref  Close to zero^c^  1.98 (0.70,5.59) | Ref  Close to zero^d^  1.77 (0.62,5.08) |
| **Adulthood SES**  Low  Middle  High | Ref  0.85 (0.59,1.22)  0.72 (0.46,1.11) | Ref  0.93 (0.65,1.34)  0.79 (0.50,1.23) | Ref  1.05 (0.61,1.81)  0.84 (0.42,1.70) | Ref  1.15 (0.67,1.99)  0.95 (0.47,1.94) | Ref  0.70 (0.41,1.22)  0.54 (0.27,1.09) | Ref  0.76 (0.43,1.31)  0.56 (0.28,1.14) | Ref  0.93 (0.29,2.93)  1.10 (0.34,3.53) | Ref  1.27 (0.41,3.88)  1.70 (0.55,5.22) |
| **Social mobility**  Stable low SES  Downward mobility  Upward mobility  Stable middle or high SES | Ref  1.76 (1.02,3.02)  1.08 (0.60,1.95)  1.19 (0.70,2.00) | Ref  1.54 (0.90,2.64)  1.18 (0.65,2.13)  1.16 (0.69,1.95) | Ref  1.88 (0.57,6.22)  1.12 (0.23,5.53)  1.77 (0.54,5.79) | Ref  1.75 (0.54,5.74)  1.08 (0.22,5.32)  1.86 (0.57,6.05) | Ref  2.27 (1.12,4.61)  1.21 (0.57,2.57)  0.90 (0.42,1.93) | Ref  1.94 (0.97,3.89)  1.31 (0.61,2.80)  0.82 (0.38,1.77) | Ref  Close to zero^e^  0.75 (0.25,2.23)  0.98 (0.28,3.45) | Ref  Close to zero^f^  1.02 (0.36,2.93)  1.15 (0.35,3.71) |
| ***(C) Colorectal cancer*** |  | |  | |  | |  | |
| **Childhood SES**  Low  Middle  High | Ref  0.68 (0.42,1.12)  0.49 (0.27,0.90) | Ref  0.67 (0.41,1.09)  0.48 (0.26,0.88) | Ref  0.66 (0.29,1.53)  0.47 (0.16,1.42) | Ref  0.65 (0.28,1.48)  0.46 (0.15,1.38) | Ref  0.72 (0.33,1.58)  0.65 (0.26,1.60) | Ref  0.71 (0.32,1.56)  0.62 (0.26,1.51) | Ref  0.70 (0.25,1.92)  0.30 (0.07,1.34) | Ref  0.71 (0.27,1.89)  0.31 (0.07,1.39) |
| **Adulthood SES**  Low  Middle  High | Ref  1.38 (0.88,2.14)  1.16 (0.69,1.94) | Ref  1.37 (0.87,2.14)  1.16 (0.69,1.94) | Ref  1.33 (0.63,2.84)  1.81 (0.76,4.31) | Ref  1.36 (0.64,2.89)  1.86 (0.80,4.31) | Ref  1.43 (0.74,2.77)  0.57 (0.19,1.68) | Ref  1.41 (0.72,2.78)  0.54 (0.18,1.68) | Ref  1.34 (0.50,3.57)  1.20 (0.46,3.15) | Ref  1.37 (0.52,3.65)  1.31 (0.51,3.37) |
| **Social mobility**  Stable low SES  Downward mobility  Upward mobility  Stable middle or high SES | Ref  0.67 (0.33,1.34)  1.49 (0.84,2.66)  0.81 (0.44,1.50) | Ref  0.66 (0.33,1.30)  1.48 (0.83,2.66)  0.80 (0.43,1.47) | Ref  0.69 (0.19,2.56)  1.89 (0.45,7.93)  1.00 (0.29,3.50) | Ref  0.67 (0.18,2.47)  1.92 (0.46,8.09)  1.00 (0.28,3.51) | Ref  0.84 (0.32,2.24)  1.36 (0.59,3.16)  0.78 (0.30,2.01) | Ref  0.82 (0.31,2.15)  1.34 (0.57,3.16)  0.75 (0.28,1.97) | Ref  0.65 (0.08,5.49)  1.43 (0.53,3.83)  0.65 (0.19,2.18) | Ref  0.65 (0.08,5.61)  1.51 (0.57,4.00)  0.69 (0.21,2.29) |

a: Minimally adjusted model: adjusted for age and country.

b: Fully adjusted model: additionally adjusted for marital status, smoking status, and daily alcohol consumption.

c: Sub-HR=9.63×10^-7^, 95% CI: 5.56×10^-7^, 1.67×10^-6^.

d: Sub-HR= 3.09×10^-7^, 95% CI: 1.57×10^-7^, 6.08×10^-7^.

e: Sub-HR=8.66×10^-10^, 95% CI: 3.50×10^-10^, 2.14×10^-9^.

f: Sub-HR= 4.57×10^-8^, 95% CI: 1.62×10^-8^, 1.29×10^-7^.

SES: socioeconomic status.

**Table S8** Crude cancer mortality rates per 1000 person-years by socioeconomic status and social mobility groups in Central and Eastern Europe: results stratified by sex

|  | **Cancer mortality rates per 1000 person-years** | | | | | | | | | | | |
| --- | --- | --- | --- | --- | --- | --- | --- | --- | --- | --- | --- | --- |
|  | **Total** | | | | **Lung** | | | | **Colorectal** | | | |
|  | **All** | **CZ** | **PL** | **LT** | **All** | **CZ** | **PL** | **LT** | **All** | **CZ** | **PL** | **LT** |
| ***Males*** |  |  |  |  |  |  |  |  |  |  |  |  |
| **Childhood SES** |  |  |  |  |  |  |  |  |  |  |  |  |
| Low | 9.98 | 11.11 | 9.72 | 9.93 | 2.28 | 2.33 | 2.38 | 2.14 | 1.42 | 1.94 | 1.33 | 1.36 |
| Middle | 8.08 | 8.68 | 7.46 | 7.27 | 1.95 | 1.78 | 2.40 | 1.34 | 1.06 | 1.41 | 0.72 | 0.59 |
| High | 6.11 | 5.71 | 6.68 | 5.97 | 1.73 | 1.67 | 2.03 | 1.26 | 0.82 | 0.74 | 0.92 | 0.84 |
| **Adulthood SES** |  |  |  |  |  |  |  |  |  |  |  |  |
| Low | 9.59 | 9.02 | 9.59 | 12.16 | 2.60 | 2.35 | 2.74 | 3.04 | 1.39 | 1.53 | 1.12 | 2.17 |
| Middle | 9.40 | 8.99 | 9.20 | 10.34 | 2.23 | 1.45 | 2.72 | 2.54 | 1.27 | 1.23 | 1.29 | 1.31 |
| High | 6.20 | 5.74 | 5.94 | 6.95 | 1.41 | 1.59 | 1.46 | 1.18 | 0.80 | 0.88 | 0.73 | 0.80 |
| **Social mobility** |  |  |  |  |  |  |  |  |  |  |  |  |
| Stable low SES | 11.80 | 10.41 | 11.79 | 13.26 | 3.01 | 2.84 | 2.90 | 3.56 | 1.71 | 2.21 | 1.50 | 1.94 |
| Downward mobility | 8.44 | 8.77 | 7.84 | 9.92 | 2.39 | 2.26 | 2.61 | 1.98 | 1.23 | 1.41 | 0.82 | 2.64 |
| Upward mobility | 9.29 | 11.59 | 8.54 | 9.45 | 2.00 | 1.97 | 2.08 | 1.94 | 1.30 | 1.75 | 1.23 | 1.28 |
| Stable middle or high SES | 6.53 | 6.58 | 6.67 | 6.16 | 1.61 | 1.48 | 2.01 | 1.22 | 0.82 | 0.94 | 0.82 | 0.54 |
| **Total** | 8.12 | 7.76 | 8.18 | 8.56 | 2.00 | 1.80 | 2.28 | 1.80 | 1.11 | 1.20 | 1.03 | 1.11 |
| ***Females*** |  |  |  |  |  |  |  |  |  |  |  |  |
| **Childhood SES** |  |  |  |  |  |  |  |  |  |  |  |  |
| Low | 5.21 | 7.48 | 5.37 | 4.51 | 0.62 | 0.74 | 0.81 | 0.40 | 0.71 | 0.98 | 0.66 | 0.69 |
| Middle | 4.70 | 4.67 | 4.76 | 4.68 | 0.83 | 0.92 | 1.01 | 0 | 0.49 | 0.54 | 0.41 | 0.45 |
| High | 3.98 | 3.89 | 3.98 | 4.19 | 0.73 | 0.76 | 0.82 | 0.46 | 0.27 | 0.28 | 0.29 | 0.18 |
| **Adulthood SES** |  |  |  |  |  |  |  |  |  |  |  |  |
| Low | 5.04 | 4.94 | 5.09 | 5.15 | 0.90 | 0.86 | 1.09 | 0.41 | 0.44 | 0.38 | 0.46 | 0.49 |
| Middle | 5.25 | 5.09 | 5.26 | 5.42 | 0.76 | 1.01 | 0.81 | 0.38 | 0.66 | 0.59 | 0.72 | 0.66 |
| High | 3.52 | 3.59 | 3.60 | 3.41 | 0.45 | 0.57 | 0.52 | 0.29 | 0.42 | 0.52 | 0.21 | 0.50 |
| **Social mobility** |  |  |  |  |  |  |  |  |  |  |  |  |
| Stable low SES | 5.56 | 6.96 | 5.53 | 4.96 | 0.68 | 0.70 | 0.75 | 0.55 | 0.59 | 0.70 | 0.58 | 0.55 |
| Downward mobility | 4.72 | 4.65 | 4.68 | 5.69 | 1.02 | 0.88 | 1.40 | 0 | 0.35 | 0.34 | 0.36 | 0.32 |
| Upward mobility | 4.96 | 8.07 | 5.19 | 4.35 | 0.58 | 0.78 | 0.89 | 0.35 | 0.80 | 1.30 | 0.76 | 0.74 |
| Stable middle or high SES | 4.10 | 4.06 | 4.12 | 4.17 | 0.63 | 0.82 | 0.56 | 0.30 | 0.40 | 0.49 | 0.33 | 0.30 |
| **Total** | 4.65 | 4.65 | 4.78 | 4.47 | 0.72 | 0.83 | 0.87 | 0.35 | 0.50 | 0.48 | 0.48 | 0.55 |

CZ: Czech Republic, PL: Poland, LT: Lithuania, SES: socioeconomic status.

**Table S9** Age-standardised cancer mortality rates per 1000 person-years by socioeconomic status and social mobility groups in Central and Eastern Europe: results stratified by sex

|  | **Age-standardised cancer mortality rates per 1000 person-years** | | | | | | | | | | | |
| --- | --- | --- | --- | --- | --- | --- | --- | --- | --- | --- | --- | --- |
|  | **Total** | | | | **Lung** | | | | **Colorectal** | | | |
|  | **All** | **CZ** | **PL** | **LT** | **All** | **CZ** | **PL** | **LT** | **All** | **CZ** | **PL** | **LT** |
| ***Males*** |  |  |  |  |  |  |  |  |  |  |  |  |
| **Childhood SES** |  |  |  |  |  |  |  |  |  |  |  |  |
| Low | 8.84 | 8.60 | 9.83 | 8.38 | 1.96 | 1.35 | 2.25 | 1.97 | 1.31 | 1.28 | 1.50 | 0.98 |
| Middle | 8.39 | 8.07 | 10.18 | 7.21 | 2.03 | 1.90 | 3.40 | 1.36 | 1.10 | 1.39 | 0.97 | 0.56 |
| High | 9.42 | 8.88 | 11.56 | 7.60 | 2.47 | 3.46 | 2.93 | 1.41 | 1.26 | 0.86 | 0.91 | 1.31 |
| **Adulthood SES** |  |  |  |  |  |  |  |  |  |  |  |  |
| Low | 10.50 | 10.22 | 11.10 | 11.26 | 2.79 | 2.78 | 2.97 | 2.84 | 1.79 | 2.04 | 1.57 | 1.79 |
| Middle | 8.51 | 8.39 | 10.46 | 7.95 | 1.97 | 1.14 | 2.78 | 2.16 | 1.25 | 1.26 | 1.39 | 0.85 |
| High | 6.98 | 6.61 | 7.83 | 6.76 | 1.56 | 1.93 | 2.34 | 1.12 | 0.86 | 1.10 | 0.66 | 0.73 |
| **Social mobility** |  |  |  |  |  |  |  |  |  |  |  |  |
| Stable low SES | 10.98 | 7.74 | 11.73 | 12.38 | 2.62 | 1.80 | 2.56 | 3.19 | 1.68 | 1.54 | 1.66 | 1.52 |
| Downward mobility | 10.74 | 11.36 | 10.65 | 10.81 | 2.98 | 3.34 | 3.20 | 2.15 | 2.14 | 2.12 | 1.75 | 2.90 |
| Upward mobility | 7.95 | 8.59 | 8.58 | 7.72 | 1.72 | 0.95 | 2.07 | 1.77 | 1.14 | 1.10 | 1.35 | 0.90 |
| Stable middle or high SES | 7.95 | 7.06 | 10.66 | 6.85 | 1.88 | 1.81 | 3.23 | 1.25 | 0.89 | 0.99 | 0.75 | 0.62 |
| **Total** | 8.54 | 8.46 | 9.95 | 7.74 | 2.05 | 1.93 | 2.73 | 1.67 | 1.25 | 1.46 | 1.24 | 0.97 |
| ***Females*** |  |  |  |  |  |  |  |  |  |  |  |  |
| **Childhood SES** |  |  |  |  |  |  |  |  |  |  |  |  |
| Low | 4.79 | 5.41 | 5.69 | 3.84 | 0.60 | 0.61 | 0.72 | 0.29 | 0.65 | 0.55 | 0.90 | 0.62 |
| Middle | 4.81 | 4.25 | 4.72 | 5.03 | 0.71 | 0.77 | 0.87 | 0 | 0.49 | 0.51 | 0.64 | 0.41 |
| High | 5.15 | 4.28 | 4.80 | 4.99 | 0.85 | 0.75 | 1.04 | 0.55 | 0.31 | 0.35 | 0.34 | 0.20 |
| **Adulthood SES** |  |  |  |  |  |  |  |  |  |  |  |  |
| Low | 5.16 | 5.26 | 5.38 | 4.38 | 0.89 | 1.08 | 0.96 | 0.29 | 0.43 | 0.33 | 0.56 | 0.39 |
| Middle | 4.94 | 4.46 | 5.61 | 4.66 | 0.65 | 0.65 | 0.67 | 0.30 | 0.62 | 0.38 | 1.12 | 0.52 |
| High | 3.89 | 4.29 | 3.34 | 3.45 | 0.49 | 0.55 | 0.51 | 0.29 | 0.48 | 1.34 | 0.17 | 0.51 |
| **Social mobility** |  |  |  |  |  |  |  |  |  |  |  |  |
| Stable low SES | 5.04 | 4.81 | 5.22 | 3.81 | 0.66 | 0.92 | 0.66 | 0.35 | 0.46 | 0.42 | 0.44 | 0.37 |
| Downward mobility | 5.45 | 5.10 | 6.28 | 5.86 | 1.07 | 1.04 | 1.46 | 0 | 0.42 | 0.29 | 0.88 | 0.31 |
| Upward mobility | 4.62 | 6.13 | 6.42 | 3.90 | 0.57 | 0.34 | 0.80 | 0.27 | 0.78 | 0.58 | 1.55 | 0.72 |
| Stable middle or high SES | 4.62 | 4.33 | 3.86 | 4.81 | 0.56 | 0.68 | 0.50 | 0.30 | 0.42 | 0.63 | 0.35 | 0.28 |
| **Total** | 4.82 | 4.96 | 5.16 | 4.08 | 0.70 | 0.86 | 0.76 | 0.29 | 0.53 | 0.48 | 0.72 | 0.51 |

CZ: Czech Republic, PL: Poland, LT: Lithuania, SES: socioeconomic status.

**Table S10** Crude cancer mortality rates per 1000 person-years by socioeconomic status and social mobility groups in Central and Eastern European countries

|  | **Cancer mortality rates per 1000 person-years** | | | | | | | | | | | |
| --- | --- | --- | --- | --- | --- | --- | --- | --- | --- | --- | --- | --- |
|  | **Total** | | | | **Lung** | | | | **Colorectal** | | | |
|  | **All** | **CZ** | **PL** | **LT** | **All** | **CZ** | **PL** | **LT** | **All** | **CZ** | **PL** | **LT** |
| **Childhood SES** |  |  |  |  |  |  |  |  |  |  |  |  |
| Low | 7.34 | 9.25 | 7.42 | 6.75 | 1.36 | 1.51 | 1.55 | 1.12 | 1.03 | 1.45 | 0.98 | 0.97 |
| Middle | 6.22 | 6.44 | 6.04 | 5.79 | 1.33 | 1.30 | 1.67 | 0.57 | 0.75 | 0.92 | 0.56 | 0.51 |
| High | 4.95 | 4.71 | 5.22 | 5.02 | 1.19 | 1.17 | 1.38 | 0.83 | 0.52 | 0.49 | 0.58 | 0.49 |
| **Adulthood SES** |  |  |  |  |  |  |  |  |  |  |  |  |
| Low | 6.74 | 6.50 | 6.88 | 7.06 | 1.54 | 1.43 | 1.75 | 1.13 | 0.79 | 0.82 | 0.73 | 0.95 |
| Middle | 7.06 | 6.77 | 7.11 | 7.39 | 1.41 | 1.20 | 1.71 | 1.25 | 0.93 | 0.86 | 0.99 | 0.92 |
| High | 4.96 | 4.78 | 4.92 | 5.17 | 0.97 | 1.14 | 1.05 | 0.73 | 0.62 | 0.72 | 0.50 | 0.65 |
| **Social mobility** |  |  |  |  |  |  |  |  |  |  |  |  |
| Stable low SES | 7.78 | 8.42 | 7.92 | 7.07 | 1.51 | 1.60 | 1.57 | 1.31 | 0.99 | 1.34 | 0.93 | 0.90 |
| Downward mobility | 6.16 | 6.19 | 5.98 | 7.05 | 1.55 | 1.40 | 1.90 | 0.64 | 0.69 | 0.74 | 0.55 | 1.07 |
| Upward mobility | 7.11 | 9.98 | 7.01 | 6.66 | 1.28 | 1.43 | 1.54 | 1.07 | 1.05 | 1.55 | 1.01 | 0.99 |
| Stable middle or high SES | 5.29 | 5.29 | 5.39 | 5.10 | 1.11 | 1.14 | 1.28 | 0.73 | 0.61 | 0.71 | 0.58 | 0.41 |
| **Total** | 6.22 | 6.05 | 6.37 | 6.22 | 1.30 | 1.27 | 1.53 | 0.97 | 0.77 | 0.80 | 0.74 | 0.79 |

CZ: Czech Republic, PL: Poland, LT: Lithuania, SES: socioeconomic status.

**Table S11** Baseline characteristics of the analytical sample and the excluded participants

| **Sample characteristics** | | **Analytical sample** | **Excluded participants^a^** | **P-values^b^** |
| --- | --- | --- | --- | --- |
|  |  | n=23009 (col %) | n=3737 (col %) |  |
| Age (year) | Mean (SD) | 58.63 (7.35) | 59.48 (0.12) | <0.001 |
| Sex | Male  Female | 10899 (47.37)  12110 (52.63) | 1708 (45.71)  2029 (54.29) | 0.06 |
| Marital status | Single  Married/cohabiting  Divorced/separated/widowed | 919 (3.99)  17240 (74.93)  4850 (21.08) | 191 (5.32)  2524 (70.33)  874 (24.35) | <0.001 |
| Smoking status | Never smoker  Former smoker  Current smoker | 10859 (47.19)  6021 (26.17)  6129 (26.64) | 1668 (47.68)  853 (24.39)  977 (27.93) | 0.06 |
| Daily alcohol consumption (g/day) | Mean (SD) | 162.97 (367.15) | 162.49 (380.81) | 0.94 |

a: Numbers may not total 3,737 due to missing data in covariates.

b: P-values for differences between the analytical sample and the excluded participants.

SD: standard deviation.

**Table S12** Associations of childhood SES measured by household amenities at age 10, and social mobility with cancer mortality in Central and Eastern Europe: subdistribution hazard ratios and 95% confidence intervals

|  | **Subdistribution hazard ratios (95% confidence intervals)** | | | | | | | |
| --- | --- | --- | --- | --- | --- | --- | --- | --- |
|  | **All countries**  n=23009 | | **Czech Republic**  n=7545 | | **Poland**  n=9252 | | **Lithuania**  n=6212 | |
|  | Minimally adjusted^a^ | Fully adjusted^b^ | Age-sex adjusted | Fully adjusted^b^ | Age-sex adjusted | Fully adjusted^b^ | Age-sex adjusted | Fully adjusted^b^ |
| ***(A) Total cancer*** |  | |  | |  | |  | |
| **Childhood SES**  Low  Middle  High | Ref  1.05 (0.95,1.16)  1.08 (0.95,1.22) | Ref  1.04 (0.94,1.15)  1.05 (0.93,1.19) | Ref  0.91 (0.75,1.11)  1.13 (0.90,1.41) | Ref  0.90 (0.74,1.09)  1.11 (0.89,1.38) | Ref  1.20 (1.03,1.39)  1.06 (0.89,1.27) | Ref  1.17 (1.01,1.36)  1.03 (0.86,1.22) | Ref  1.02 (0.85,1.21)  0.86 (0.59,1.25) | Ref  1.02 (0.85,1.22)  0.85 (0.58,1.24) |
| **Social mobility**  Stable low SES  Downward mobility  Upward mobility  Stable middle/high SES | Ref  1.08 (0.94,1.25)  0.84 (0.73,0.97)  0.90 (0.79,1.03) | Ref  1.04 (0.90,1.20)  0.88 (0.76,1.02)  0.93 (0.82,1.07) | Ref  1.13 (0.86,1.50)  1.06 (0.76,1.49)  0.92 (0.70,1.21) | Ref  1.09 (0.82,1.44)  1.10 (0.79,1.54)  0.94 (0.72,1.24) | Ref  1.16 (0.95,1.41)  0.81 (0.66,0.99)  0.94 (0.78,1.13) | Ref  1.11 (0.91,1.36)  0.87 (0.71,1.06)  0.99 (0.82,1.19) | Ref  0.80 (0.55,1.15)  0.73 (0.55,0.97)  0.78 (0.59,1.04) | Ref  0.78 (0.54,1.13)  0.74 (0.56,0.99)  0.81 (0.60,1.08) |
| ***(B) Lung cancer*** |  | |  | |  | |  | |
| **Childhood SES**  Low  Middle  High | Ref  1.08 (0.87,1.35)  1.29 (0.99,1.67) | Ref  1.03 (0.82,1.28)  1.20 (0.92,1.56) | Ref  1.13 (0.72,1.79)  1.59 (0.95,2.68) | Ref  1.07 (0.68,1.70)  1.52 (0.91,2.56) | Ref  1.14 (0.83,1.56)  1.18 (0.83,1.67) | Ref  1.08 (0.79,1.49)  1.08 (0.76,1.53) | Ref  1.05 (0.67,1.64)  1.02 (0.40,2.57) | Ref  1.07 (0.68,1.69)  1.02 (0.40,2.57) |
| **Social mobility**  Stable low SES  Downward mobility  Upward mobility  Stable middle/high SES | Ref  1.21 (0.89,1.63)  0.75 (0.54,1.04)  0.86 (0.65,1.15) | Ref  1.10 (0.81,1.49)  0.85 (0.61,1.18)  0.93 (0.69,1.24) | Ref  1.26 (0.67,2.35)  0.76 (0.34,1.71)  0.96 (0.52,1.78) | Ref  1.15 (0.61,2.14)  0.82 (0.36,1.86)  1.01 (0.55,1.87) | Ref  1.37 (0.92,2.04)  0.82 (0.55,1.24)  0.87 (0.59,1.28) | Ref  1.27 (0.85,1.89)  0.94 (0.62,1.42)  0.92 (0.63,1.36) | Ref  0.55 (0.22,1.39)  0.45(0.23,0.90)  0.57 (0.29,1.13) | Ref  0.48 (0.19,1.22)  0.53 (0.27,1.06)  0.71 (0.36,1.40) |
| ***(C) Colorectal cancer*** |  | |  | |  | |  | |
| **Childhood SES**  Low  Middle  High | Ref  0.71 (0.55,0.93)  0.78 (0.55,1.10) | Ref  0.71 (0.55,0.93)  0.78 (0.55,1.11) | Ref  0.88 (0.54,1.43)  0.80 (0.45,1.43) | Ref  0.88 (0.54,1.43)  0.81 (0.45,1.43) | Ref  0.61 (0.38,0.98)  0.79 (0.48,1.30) | Ref  0.61 (0.37,0.98)  0.79 (0.48,1.29) | Ref  0.64 (0.40,1.04)  1.01 (0.38,2.67) | Ref  0.65 (0.40,1.06)  1.07 (0.40,2.86) |
| **Social mobility**  Stable low SES  Downward mobility  Upward mobility  Stable middle/high SES | Ref  0.81 (0.54,1.22)  1.00 (0.68,1.46)  0.70 (0.48,1.01) | Ref  0.80 (0.54,1.21)  0.99 (0.67,1.45)  0.69 (0.48,1.00) | Ref  0.91 (0.45,1.84)  0.95 (0.41,2.19)  0.80 (0.41,1.59) | Ref  0.88 (0.43,1.77)  0.93 (0.40,2.16)  0.80 (0.40,1.59) | Ref  0.82 (0.44,1.53)  1.12 (0.67,1.90)  0.69 (0.40,1.18) | Ref  0.82 (0.44,1.53)  1.15 (0.68,1.96)  0.70 (0.40,1.21) | Ref  0.65 (0.23,1.87)  0.81 (0.39,1.68)  0.55 (0.26,1.19) | Ref  0.65 (0.23,1.88)  0.80 (0.39,1.67)  0.56 (0.26,1.20) |

a: Minimally adjusted model: adjusted for age, sex, and country.

b: Fully adjusted model: additionally adjusted for marital status, smoking status, and daily alcohol consumption.

SES: socioeconomic status.

**Table S13** Associations of childhood SES measured by both parental education and household amenities at age 10, and social mobility with cancer mortality in Central and Eastern Europe: subdistribution hazard ratios and 95% confidence intervals

|  | **Subdistribution hazard ratios (95% confidence intervals)** | | | | | | | |
| --- | --- | --- | --- | --- | --- | --- | --- | --- |
|  | **All countries**  n=19855 | | **Czech Republic**  n=4391 | | **Poland**  n=9252 | | **Lithuania**  n=6212 | |
|  | Minimally adjusted^a^ | Fully adjusted^b^ | Age-sex adjusted | Fully adjusted^b^ | Age-sex adjusted | Fully adjusted^b^ | Age-sex adjusted | Fully adjusted^b^ |
| ***(A) Total cancer*** |  | |  | |  | |  | |
| **Childhood SES**  Low  Middle  High | Ref  0.98 (0.88,1.10)  1.03 (0.91,1.16) | Ref  0.95 (0.85,1.06)  0.99 (0.87,1.12) | Ref  0.95 (0.75,1.20)  0.99 (0.75,1.31) | Ref  0.94 (0.74,1.19)  0.98 (0.74,1.29) | Ref  0.96 (0.82,1.13)  1.03 (0.87,1.22) | Ref  0.92 (0.79,1.07)  0.97 (0.82,1.15) | Ref  1.02 (0.81,1.29)  1.05 (0.83,1.32) | Ref  1.00 (0.79,1.26)  1.04 (0.82,1.31) |
| **Social mobility**  Stable low SES  Downward mobility  Upward mobility  Stable middle/high SES | Ref  1.05 (0.90,1.23)  0.88 (0.77,1.01)  0.87 (0.76,1.00) | Ref  0.99 (0.85,1.15)  0.92 (0.80,1.05)  0.89 (0.78,1.02) | Ref  1.19 (0.83,1.71)  1.19 (0.80,1.77)  1.01 (0.71,1.42) | Ref  1.14 (0.80,1.64)  1.20 (0.81,1.79)  1.02 (0.72,1.44) | Ref  1.00 (0.82,1.22)  0.82 (0.68,0.98)  0.83 (0.69,0.99) | Ref  0.93 (0.76,1.13)  0.87 (0.72,1.05)  0.85 (0.71,1.01) | Ref  1.22 (0.82,1.84)  0.89 (0.69,1.13)  0.90 (0.68,1.19) | Ref  1.17 (0.78,1.76)  0.92 (0.72,1.17)  0.91 (0.69,1.21) |
| ***(B) Lung cancer*** |  | |  | |  | |  | |
| **Childhood SES**  Low  Middle  High | Ref  1.14 (0.90,1.45)  1.23 (0.95,1.59) | Ref  1.03 (0.81,1.31)  1.11 (0.86,1.43) | Ref  1.51 (0.82,2.77)  1.66 (0.84,3.27) | Ref  1.43 (0.77,2.62)  1.54 (0.78,3.04) | Ref  1.27 (0.93,1.74)  1.26 (0.90,1.78) | Ref  1.13 (0.83,1.55)  1.12 (0.80,1.57) | Ref  0.59 (0.29,1.17)  0.99 (0.56,1.76) | Ref  0.54 (0.27,1.08)  0.96 (0.55,1.70) |
| **Social mobility**  Stable low SES  Downward mobility  Upward mobility  Stable middle/high SES | Ref  1.31 (0.95,1.81)  0.81 (0.59,1.11)  0.92 (0.68,1.25) | Ref  1.12 (0.81,1.55)  0.92 (0.67,1.26)  0.96 (0.71,1.29) | Ref  1.15 (0.52,2.56)  0.54 (0.19,1.56)  1.14 (0.53,2.42) | Ref  1.04 (0.47,2.30)  0.61 (0.21,1.73)  1.21 (0.57,2.54) | Ref  1.57 (1.06,2.35)  0.86 (0.57,1.28)  0.95 (0.64,1.41) | Ref  1.34 (0.90,2.00)  0.96 (0.64,1.44)  0.96 (0.65,1.41) | Ref  0.53 (0.16,1.81)  0.62 (0.35,1.10)  0.54 (0.28,1.06) | Ref  0.44 (0.13,1.55)  0.78 (0.44,1.39)  0.66 (0.33,1.29) |
| ***(C) Colorectal cancer*** |  | |  | |  | |  | |
| **Childhood SES**  Low  Middle  High | Ref  0.65 (0.47,0.91)  0.69 (0.48,1.00) | Ref  0.65 (0.46,0.90)  0.69 (0.48,1.00) | Ref  0.51(0.28,0.90)  0.30(0.14,0.67) | Ref  0.50 (0.28,0.90)  0.30(0.14,0.68) | Ref  0.69 (0.42,1.12)  0.89 (0.55,1.44) | Ref  0.68 (0.42,1.11)  0.88 (0.54,1.44) | Ref  0.64 (0.31,1.33)  0.77 (0.38,1.56) | Ref  0.63 (0.31,1.30)  0.76 (0.37,1.53) |
| **Social mobility**  Stable low SES  Downward mobility  Upward mobility  Stable middle/high SES | Ref  0.82 (0.52,1.30)  1.05 (0.72,1.52)  0.64 (0.43,0.96) | Ref  0.82 (0.51,1.30)  1.04 (0.71,1.51)  0.63 (0.42,0.95) | Ref  0.66 (0.27,1.65)  1.36 (0.55,3.39)  0.48 (0.20,1.16) | Ref  0.63 (0.25,1.57)  1.29 (0.51,3.26)  0.47 (0.19,1.14) | Ref  0.80 (0.43,1.49)  0.99 (0.59,1.65)  0.75 (0.44,1.29) | Ref  0.79 (0.42,1.49)  1.02 (0.60,1.72)  0.77 (0.45,1.32) | Ref  1.51 (0.52,4.37)  1.04 (0.52,2.04)  0.60 (0.27,1.36) | Ref  1.50 (0.52,4.36)  1.04 (0.53,2.05)  0.60 (0.26,1.34) |

a: Minimally adjusted model: adjusted for age, sex, and country.

b: Fully adjusted model: additionally adjusted for marital status, smoking status, and daily alcohol consumption.

SES: socioeconomic status.

**Table S14** Mediation analysis with smoking and alcohol consumption as parallel mediators: results from a generalized structural equation model

| **Pathways** | **Coefficients (95% CI)** |
| --- | --- |
| **Direct effect** |  |
| Social mobility -> smoking | -0.18 (-0.21, -0.15) |
| Social mobility -> alcohol consumption | 9.77 (5.55, 13.99) |
| Social mobility -> total cancer mortality | -0.06 (-0.10, -0.02) |
| Smoking -> total cancer mortality | 0.72 (0.62, 0.81) |
| Alcohol consumption -> total cancer mortality | 0.0001 (-0.0001, 0.0002) |
| **Indirect effect** |  |
| Social mobility -> smoking -> total cancer mortality | -0.13 (-0.15, -0.10) |
| Social mobility -> alcohol consumption -> total cancer mortality | 0.0004 (-0.001, 0.001) |
| **Total effect of social mobility on total cancer mortality** | -0.19 (-0.24, -0.14) |

Results adjusted for country, age, sex, and marital status.

**Table S15** Proportion of current smokers by socioeconomic status and social mobility in Central and Eastern European countries

|  | **Proportion of current smokers (%)** | | |
| --- | --- | --- | --- |
|  | Czech Republic | Poland | Lithuania |
| **Childhood SES** |  |  |  |
| Low | 19.32 | 27.07 | 15.44 |
| Middle | 24.45 | 33.78 | 22.20 |
| High | 31.24 | 38.34 | 24.98 |
| **Adulthood SES** |  |  |  |
| Low | 31.26 | 37.96 | 19.75 |
| Middle | 21.56 | 27.00 | 16.96 |
| High | 25.07 | 29.42 | 19.11 |
| **Social mobility** |  |  |  |
| Stable low SES | 22.80 | 31.03 | 16.69 |
| Downward mobility | 32.72 | 44.14 | 28.01 |
| Upward mobility | 16.23 | 23.75 | 15.11 |
| Stable middle or high SES | 24.15 | 31.11 | 23.10 |
| **Total** | 26.47 | 32.23 | 18.51 |
