# Supplementary figures and images for: Social mobility and cancer mortality in Central and Eastern Europe: a multicohort study
Source: Eur J Public Health. 2026 Jul 11;36(4):ckag124. doi: 10.1093/eurpub/ckag124 (PMC13356042; doi:10.1093/eurpub/ckag124)

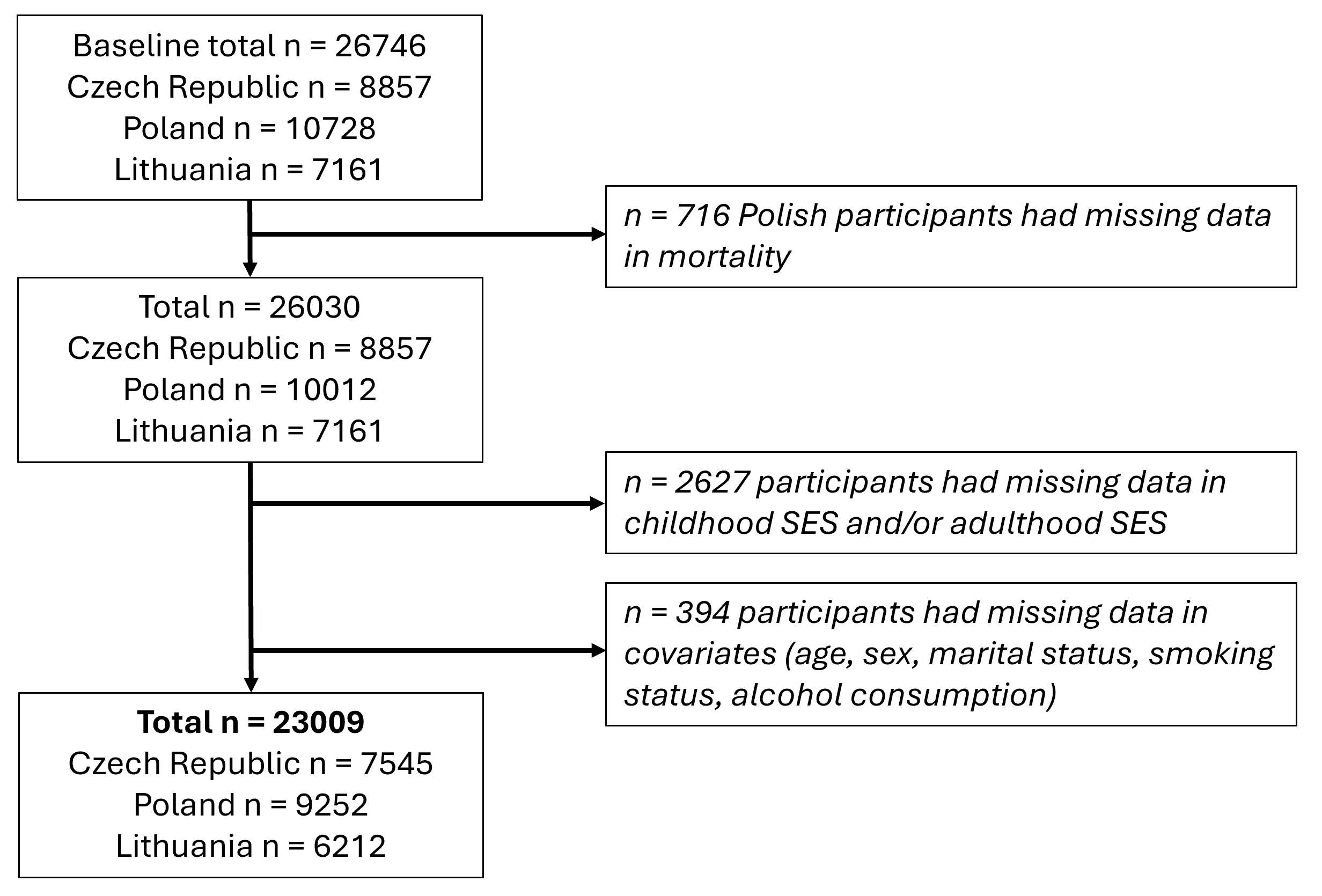

Supplement: ckag124_Supplementary_Data [file ckag124_supplementary_data.zip › ejph-2025-12-om-1051-File003.tiff]

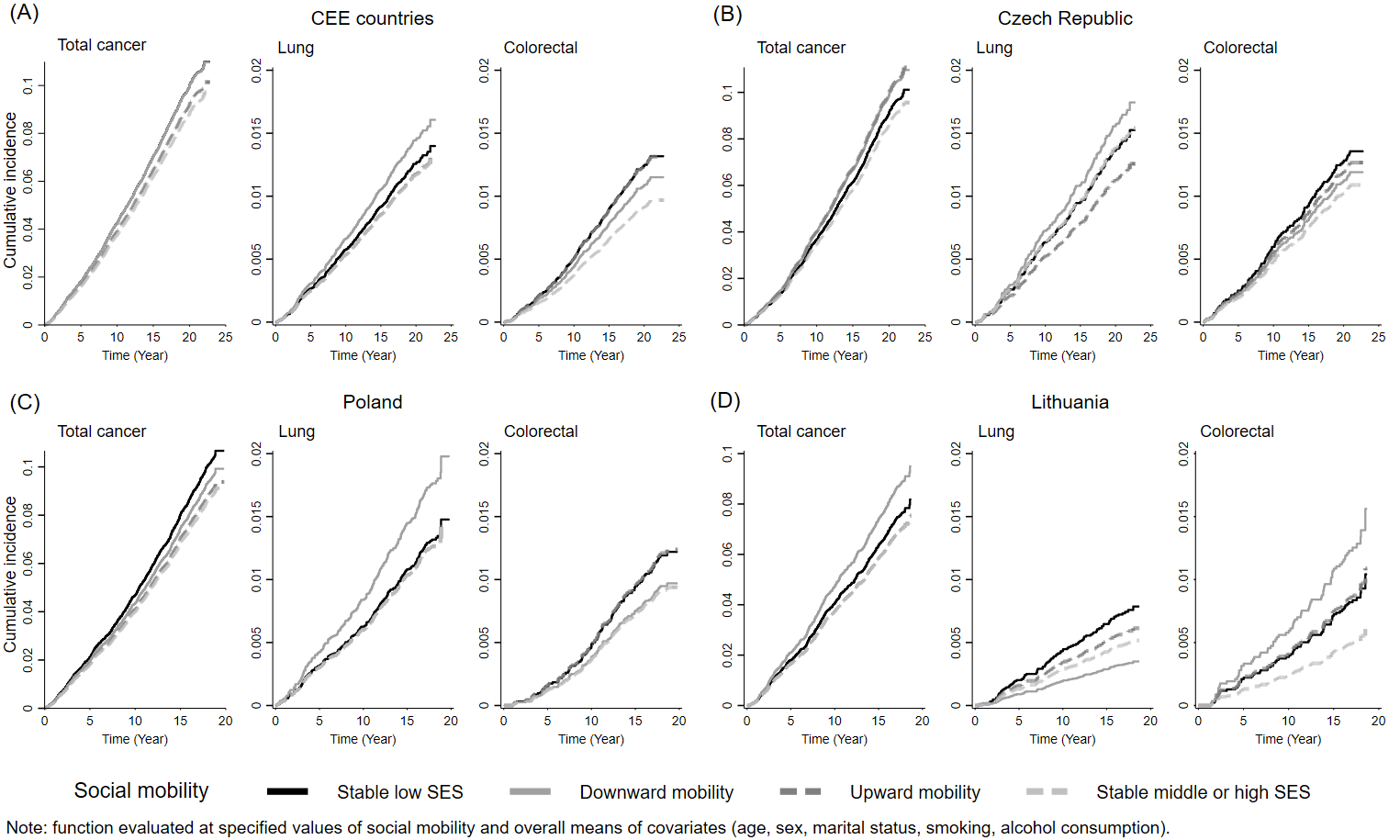

Supplement: ckag124_Supplementary_Data [file ckag124_supplementary_data.zip › ejph-2025-12-om-1051-File004.tiff]

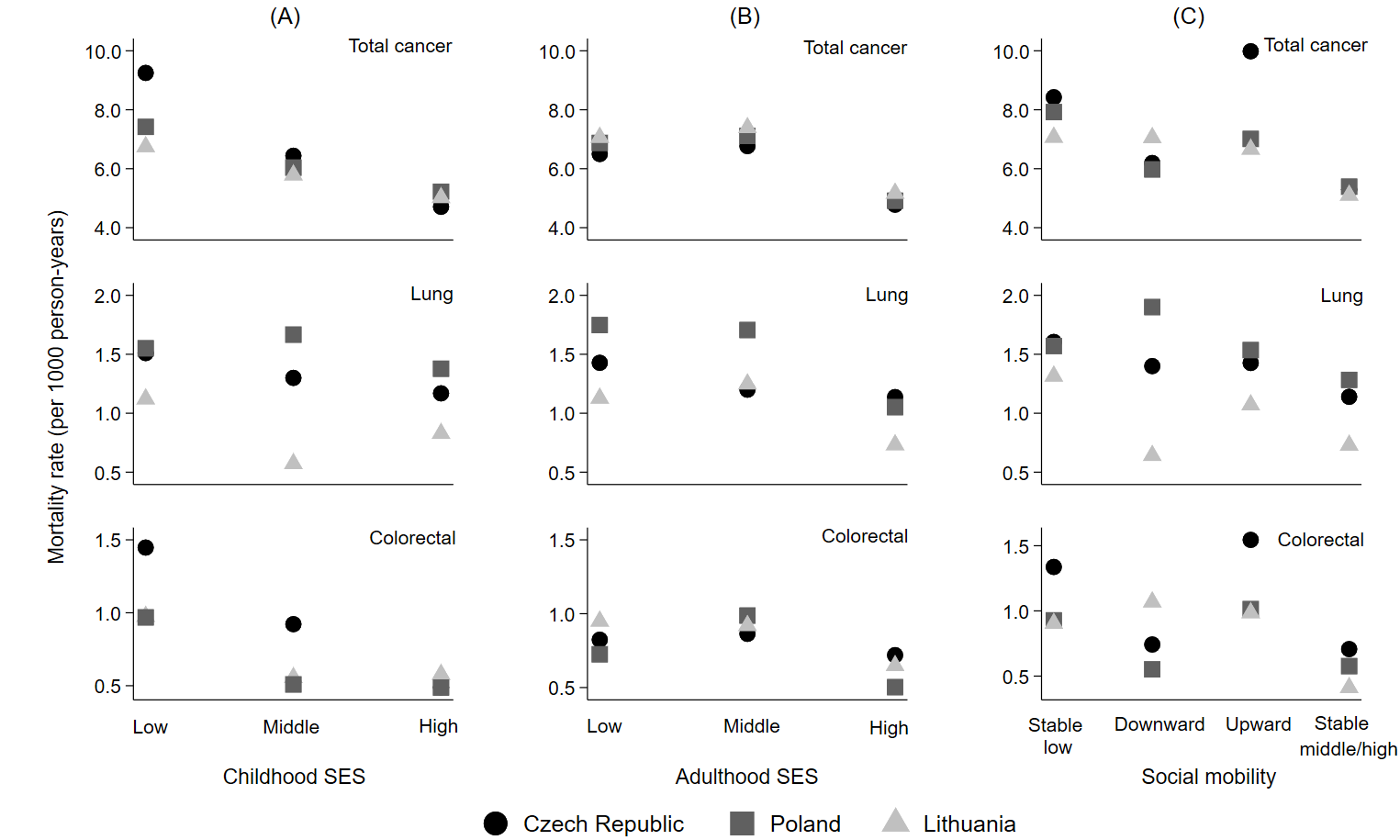

Supplement: ckag124_Supplementary_Data [file ckag124_supplementary_data.zip › ejph-2025-12-om-1051-File005.tiff]

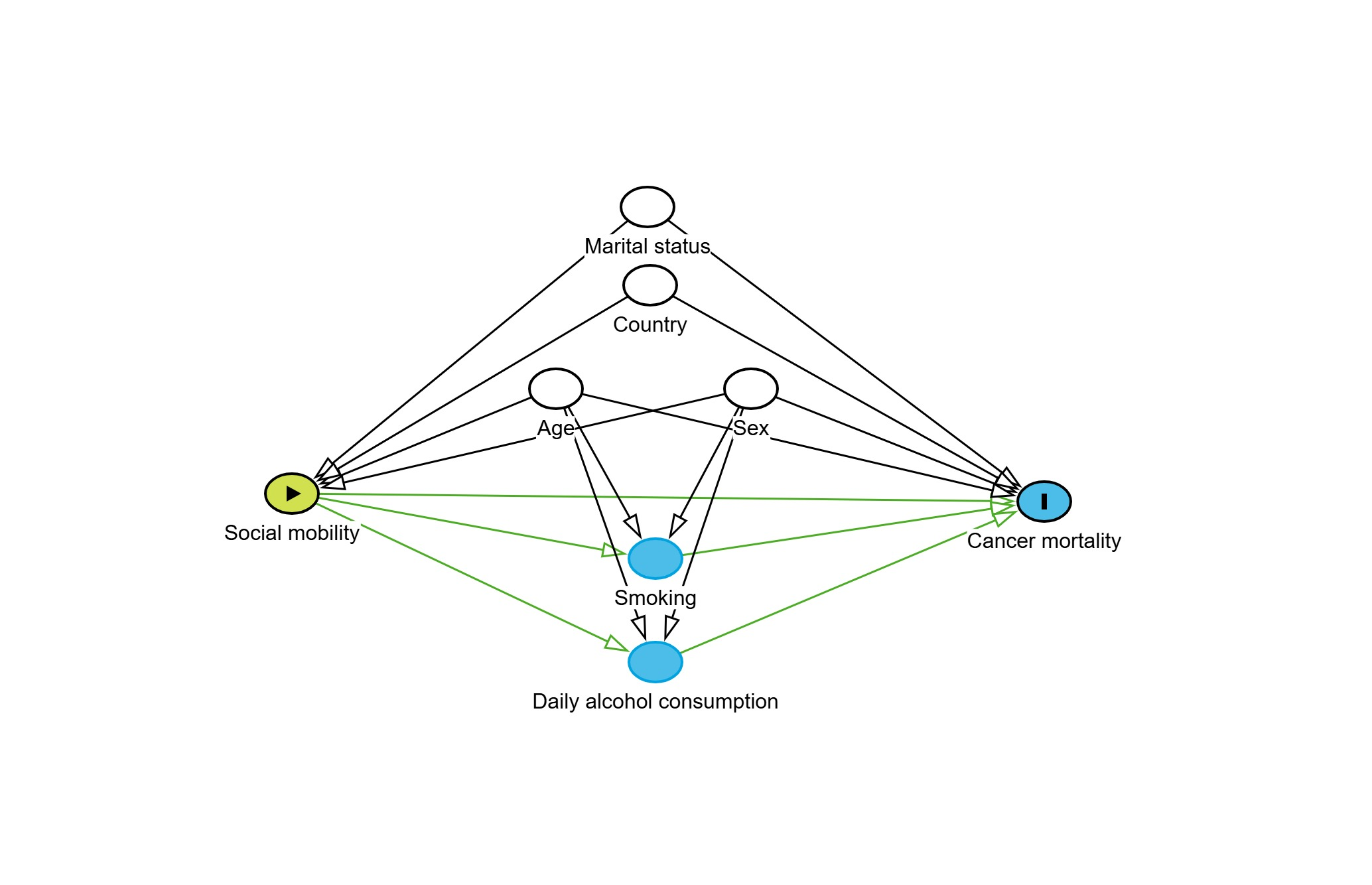

Supplement: ckag124_Supplementary_Data [file ckag124_supplementary_data.zip › ejph-2025-12-om-1051-File006.tiff]
